# Biological Evaluation of Two Magnesium Alloys from Mg-Zn-Mn-Ca and Mg-Nd-Y-Zr-Zn Systems for Bone Repair and Regeneration

**DOI:** 10.3390/jfb17090457

**Published:** 2026-09-08

**Authors:** Maria Cristina Moraru, Alexandra Iulia Dreanca, Romelia Pop, Iulian Antoniac, Aurora Antoniac, Veronica Manescu (Paltanea), Gabriel Cristescu, Gheorghe Adrian Martau, George Mihail Vlasceanu, Diana Cenariu, Marius Manole, Flaviu Alexandru Tabaran, Mariana Ionita, Dan Cristian Vodnar, Bogdan Sevastre

**Affiliations:** 1 Faculty of Veterinary Medicine, University of Agricultural Sciences and Veterinary Medicine of Cluj-Napoca, Calea Mănăştur 3-5, 400372 Cluj-Napoca, Romaniabogdan.sevastre@usamvcluj.ro (B.S.); 2 Faculty of Material Science and Engineering, National University of Science and Technology Politehnica Bucharest, 313 Splaiul Independentei, District 6, 060042 Bucharest, Romania; antoniac.iulian@gmail.com (I.A.);; 3 Academy of Romanian Scientists, 54 Splaiul Independentei, 050094 Bucharest, Romania; 4 Faculty of Electrical Engineering, National University of Science and Technology Politehnica Bucharest, 313 Splaiul Independentei, District 6, 060042 Bucharest, Romania; 5 Faculty of Food Science and Technology, University of Agricultural Sciences and Veterinary Medicine of Cluj-Napoca, Calea Mănăştur 3-5, 400372 Cluj-Napoca, Romania; 6 Institute of Life Sciences, University of Agricultural Sciences and Veterinary Medicine of Cluj-Napoca, Calea Mănăştur 3-5, 400372 Cluj-Napoca, Romania; 7 Faculty of Medical Engineering, National University of Science and Technology Politehnica Bucharest, 1-7 Gheorghe Polizu, 011061, Bucharest, Romania; george.vlasceanu@upb.ro (G.M.V.);; 8 Advanced Polymer Materials Group, National University of Science and Technology Politehnica Bucharest, 1-7 Gheorghe Polizu, 011061 Bucharest, Romania; 9 Personalized Medicine and Rare Diseases Department, MEDFUTURE-Institute for Biomedical Research, Iuliu Hațieganu University of Medicine and Pharmacy, 400349 Cluj-Napoca, Romania; diacenariu@gmail.com; 10 Department of Prosthetics and Dental Materials, Faculty of Dentistry, Iuliu Hatieganu University of Medicine and Pharmacy, 8 Victor Babes, 400012 Cluj-Napoca, Romania

**Keywords:** magnesium alloys, biodegradable biomaterials, bone regeneration, rat model, femoral condyle defect, biocompatibility

## Abstract

*Background and Objectives*: Magnesium-based biomaterials are increasingly investigated due to their biodegradability, bone-like elastic modulus, and potential osteogenic properties. However, alloy composition may influence degradation behavior and local tissue response. This study aimed to assess and compare the local biocompatibility, degradation characteristics, and bone regenerative response induced by Mg-Zn-Mn-Ca and Mg-Nd-Y-Zr-Zn magnesium alloy powders. *Materials and Methods*: Two magnesium alloys were evaluated through physicochemical characterization, in vitro osteoblast cytocompatibility and antimicrobial assays, and in vivo testing in Sprague–Dawley rats. Standardized cylindrical defects were created in the medial femoral condyle and filled with either Mg-Nd-Y-Zr-Zn or Mg-Zn-Mn-Ca alloy powders, while untreated defects served as controls. Bone remodeling and defect healing were assessed by micro-computed tomography, allowing three-dimensional qualitative and quantitative evaluation of newly formed bone. Scanning electron microscopy was used to analyze surface morphology and degradation features, while histopathological examination assessed inflammation, osteogenesis, and tissue integration at the implant site. *Results*: Both magnesium alloy powders showed antimicrobial potency and elicited no cytotoxic effects in vitro, while animal studies revealed progressive biodegradation over time, associated with new bone formation within and around the defect area. Micro-CT analysis demonstrated active bone remodeling in experimental groups, with differences in bone distribution and defect-filling patterns between Mg-Nd-Y-Zr-Zn and Mg-Zn-Mn-Ca implants. SEM revealed alloy-specific degradation morphologies. Histological evaluation showed a moderate early inflammatory response that decreased at later time points, together with ongoing osteogenesis and favorable tissue integration. No severe local adverse reactions or persistent inflammation were observed. *Conclusions*: Both Mg-Nd-Y-Zr-Zn and Mg-Zn-Mn-Ca alloy powders were locally biocompatible and supported bone regeneration in a rat femoral condyle defect model. Differences in degradation behavior and tissue response emphasize the relevance of alloy composition in developing magnesium-based biomaterials for bone defect treatment.

## 1. Introduction

The development of biodegradable metallic materials has been driven by the limitations of conventional orthopedic implants, particularly the mechanical mismatch between metallic implants and bone, which may lead to stress shielding and subsequent bone resorption [1,2]. Magnesium-based alloys are of particular interest because their elastic modulus (approximately 41–45 GPa) and density (1.74–2.0 g/cm^3^) are closer to those of cortical bone than those of titanium alloys or stainless steel [3,4,5]. In addition, magnesium is biodegradable, potentially eliminating the need for secondary implant removal. In contrast, titanium alloys and stainless steel provide high mechanical strength but have considerably higher elastic moduli and densities than bone and remain permanently in the body unless surgically removed [6,7]. These characteristics make magnesium alloys attractive for temporary orthopedic applications in which gradual degradation and mechanical compatibility with bone are required. A major limitation of magnesium alloys is their relatively rapid corrosion in physiological environments. Excessive degradation may lead to early loss of mechanical integrity and hydrogen gas accumulation before complete tissue healing [8,9]. For this reason, alloy design and surface modification have been extensively investigated to control degradation while maintaining adequate mechanical and biological properties [10,11].

The composition of magnesium alloys strongly influences their microstructure, degradation behavior, and biological response. Alloying elements can modify grain size and promote the formation of secondary intermetallic phases, which in turn affect corrosion and mechanical properties. For biomedical applications, however, alloy selection must also consider the biological effects of the released elements [12,13,14]. Rare earth elements such as Y and Nd are used to improve microstructural stability and corrosion resistance. Y promotes grain refinement and the formation of stable Mg–Zn–Y phases, while Nd can contribute to lower degradation rates and reduced hydrogen evolution [15,16,17,18,19]. Ca, Zn, and Mn are also commonly used in biodegradable magnesium alloys. Ca is an important component of bone and can improve grain refinement and mechanical properties, although excessive amounts may promote Mg_2_Ca formation and galvanic corrosion. Zn contributes to solid-solution strengthening and may improve corrosion resistance at moderate concentrations, whereas Mn can reduce the effect of metallic impurities and improve microstructural stability [20,21,22,23,24]. The suitability of magnesium alloys for bone repair cannot be determined from material characterization alone. In vitro studies provide information on cytocompatibility and antimicrobial activity, whereas in vivo models are required to assess local tissue response, degradation, and bone regeneration under physiological conditions. Combining material characterization with biological testing therefore provides a more complete evaluation of biodegradable magnesium alloys intended for bone repair [25,26].

The present study aimed to evaluate two magnesium-based alloy systems, MRI 201s and ZMX 410, as potential materials for bone defect repair. Their microstructural and phase characteristics were assessed by optical microscopy, SEM–EDS, and XRD. Biological evaluation included in vitro cytocompatibility and antimicrobial testing, followed by in vivo assessment using subcutaneous implantation and a bone defect model evaluated by histopathology and micro-computed tomography.

## 2. Materials and Methods

Two magnesium-based alloys, from the Mg-Nd-Y-Zr-Zn (MRI 201s) and Mg-Zn-Mn-Ca (ZMX 410) systems, were selected for this study. Their chemical composition is presented in Table 1. To evaluate the microstructure and degradation behavior, experimental samples were prepared as parallelepipeds with dimensions of 10 mm in length and width, and 5 mm in height [26,27,28].

### 2.1. Characterization of Experimental Samples by Microscopic Techniques and X-Ray Diffraction

The optical microscopy analysis of the experimental magnesium alloy samples was carried out using a Nikon Eclipse MA100N^®^ inverted microscope (Nikon Metrology Europe NV, Leuven, Belgium), following standard metallographic preparation procedures. These included sample mounting, grinding, polishing, and final chemical etching using a Nital solution as the etchant.

Scanning electron microscopy (SEM) investigations were performed using a Thermo Scientific™ Quattro S ESEM microscope equipped with a Thermo Scientific™ Ultra Dry™ 60 mm^2^ energy-dispersive X-ray spectroscopy (EDS) detector (Thermoxs Fisher Scientific Inc., Waltham, MA, USA), operated in High Vacuum mode at 30 kV [29,30].

Phase identification of the two investigated alloys was conducted using X-ray diffraction (XRD) on a PANALYTICAL x-Pert PRO diffractometer (Malvern Panalytical, UK). The collected data were analyzed using PDXL software version 1.2.0.1 (ICDD, 1999), which enabled the qualitative identification of the crystalline phases present in the investigated alloys.

### 2.2. In Vitro Assays

#### 2.2.1. Citotoxicity

##### Cell Culture

The osteoblast cell line was isolated and characterized from periodontal tissue, representing a primary osteogenic population (Merck KGaA, Darmstadt, Germany), as previously described by Tomuleasa et al. [31]. The cells were stained with CD44 FITC, CD49 PE, CD73 PE, CD34 FITC, CD29 PE, CD117 PE, and CD45 FITC and analyzed using a BD FACS Canto™ II flow cytometer (Becton Dickinson, Heidelberg, Germany) equipped with two lasers. Data acquisition and analysis were performed using BD FACSDiva™ software, version 6.1.3. The osteoblast cell line obtained was expanded in Gibco™ DMEM/F-12 medium containing 4.5 g/L glucose (Thermo Fisher Scientific, Waltham, MA, USA) supplemented with: 15% (*v*/*v*) standard fetal bovine serum (FBS, Thermo Fisher Scientific, Waltham, MA, USA) and 1% (*w*/*v*) penicillin/streptomycin (Thermo Fisher Scientific, Waltham, MA, USA) and 1% (*v*/*v*) penicillin/streptomycin (Thermo Fisher Scientific, Waltham, MA, USA); final concentration: 100 units/mL penicillin and 100 μg/mL streptomycin), 2 mM L^−1^ glutamine, 1% NEAA, 1% Sodium pyruvate, ascorbic acid phosphate (50 µg mL^−1^), dexamethasone (20 µM) and 1% beta-mercaptoethanol. Cells from passages 3–5 were utilized for all in vitro assays. The cells were cultivated for 24 h in 24 well plates with Corning^®^ Transwell inserts (Cat #3413; Merck KGaA, Darmstadt, Germany) at a density of 1 × 10^4^ cells per/well. When the cells reached a confluence of 80%, the 2 treatments with powder Mg alloy were added (MRI 201s and ZMX 410), at a concentration of 1 mg/mL Mg alloy/sample.

##### Cytotoxicity Assay (MTT)

The MTT assay was performed after 24 h, to assess changes in cell viability after the experimental magnesium alloy exposure. The Transwell inserts with powdered Mg alloy samples were placed on top of the cells (cultured at the bottom) for the osteoblasts to be in contact with the Mg alloy inside the insert. The cells continued to develop for another 24 h and after that, tetrazolium dye MTT (3-(4,5-dimethylthiazol-2-yl)-2,5-diphenyltetrazolium bromide) solution from Sigma-Aldrich (St. Louis, MI, USA) was used. The culture media was completely removed and on each well, control included, 250 µL Formazan dye were added and Transwell plates were placed in the incubator for 3 h. The Formazan solution was discarded and replaced with 300 µL of DMSO (dimethyl sulfoxide). Absorbance was measured at 570 nm using a Spark^®^ 10M multimode microplate reader (Tecan Group Ltd., Männedorf, Switzerland). Tecan identifies Spark^®^ as a registered trademark. The analysis was performed in triplicate. The negative control was the culture media, and the positive control was considered 100% viability (untreated cells). The absorbance raw values were normalized with the formula x = log(x).

#### 2.2.2. Microbial Minimal Inhibitory Concentrations

##### Microbial Standard Strains

The following standard strains were tested: *Staphylococcus aureus* subsp. aureus ATCC^®^ 29213, *Pseudomonas aeruginosa* ATCC^®^ 27853, *Escherichia coli* ATCC^®^ 25922, *Staphylococcus epidermidis* ATCC^®^ 12228, *Candida parapsilosis* ATCC^®^ 22019, *Candida albicans* DSMZ 1386, *Salmonella enterica* (*S. typhimurium*) ATCC^®^ 14028, *Aspergillus niger* ATCC 6275, and *Aspergillus bransiliensis* ATCC^®^ 16404. The strains were activated in a test tube containing 9 mL of sterile medium, TSB (tryptic soy broth), NB (nutrient broth), MHB (Muller-Hinton broth), YMB (yeast extract malt broth), and PDB (Potato Dextrose Broth). The tubes with TSB were incubated for 24 h at 37/30 °C *for Staphylococcus aureus subsp. aureus* ATCC^®^ 29213 and *Candida parapsilosis* ATCC^®^ 22019. The tubes with NB were incubated for 24 h at 37 °C for *Escherichia coli* ATCC^®^ 25922, *Staphylococcus epidermidis* ATCC^®^ 12228, and *Salmonella enterica* (*S. typhimurium*) ATCC^®^ 14028. The tubes with MHB were incubated for 24 h at 37 °C for *Pseudomonas aeruginosa* ATCC^®^ 27853. The tubes with YMB were incubated for 24 h at 30 °C for *Candida albicans* DSMZ 1386. The tubes with PDB were incubated for 48 h at 30 °C for *Aspergillus niger* ATCC^®^ 6275 and *Aspergillus bransiliensis* ATCC^®^ 16404. A loopful of inoculum was transferred to a growth agar medium. Plates were incubated for 24 h at 37 °C for bacteria and 4–6 days at 30 °C for fungi, respectively. Bacterial and fungal morphology was confirmed by optical microscopy (Nikon ECLIPSE Ci-L, Tokyo, Japan). Preparation of bacterial and fungal strain concentrations. Newly formed colonies of each strain, cultivated on the forementioned media, were transferred into sterile 9 mL of saline solution (8.5 g/L NaCl) and adjusted to match the turbidity of the McFarland 0.5 standard (10^8^ CFU/mL). To determine spore concentration, a solution containing 0.1% Tween^®^ 80 and sterile glass beads was used to detach spores from agar plates. The resulting spores and *Candida* spp. were then assessed by microscopic examination using a Thoma chamber. Then, a suspension of 10^5^ CFU/mL for bacteria and 10^6^ spore/mL for fungi was prepared to be added to each microplate well.

Determination of the minimum inhibitory concentration (MIC).

The MIC was determined using the resazurin microtiter plate-based antibacterial assay [1,2]. One 90 µL aliquot of strain-specific sterile medium was added to the wells of a 96-well microplate. Then, 100 µL of each sample was added to the first well, and serial 2-fold dilutions were prepared in the subsequent wells of each row by transferring 100 µL from one well to the next. The 100 µL surplus in the last well of the row was discarded. Then, 10 µL of inoculum (10^5^ CFU/mL and 10^6^ spores/mL) was added to all the wells. Gentamicin (0.4 mg/mL in saline solution) or Fluconazole (1.5 mg/mL in DMSO) was used as the positive control (C+), and water was the negative control (C−). The microplates were incubated for 24 h for bacteria and 48 h for fungi at 37 °C or 30 °C, and then 20 µL of sterile 0.2 mg/mL resazurin aqueous solution was added to all the wells [3]. The microplates were subjected to a subsequent 2 h incubation at 37 °C or 30 °C. After this period, the resazurin (a blue, non-fluorescent dye) was oxidized to resorufin (fluorescent pink) in wells containing viable bacterial cells. Thus, the concentration in the last well on each row that remained blue was considered to completely inhibit bacterial growth, the MIC.

### 2.3. In Vivo Assays

A total of 72 clinically healthy, 40-week-old, specific pathogen–free, female Sprague–Dawley rats were obtained from the National Institute for Medical–Military Research and Development “Cantacuzino” (Bucharest, Romania). As both tested materials were novel, no conventional control material was deemed appropriate. The animal was defined as the experimental unit. To minimize potential confounding factors, animals were randomly assigned to the experimental groups, and the order of treatments and outcome assessments was counterbalanced across groups. Animals were housed under standardized laboratory conditions with controlled temperature of 23 ± 2 °C, humidity of 50 ± 10%, light–dark cycles of 12/12 h, and light intensity (daytime) of 50 lux, and were provided ad libitum access to food and water. Cage positions were regularly rotated within the facility to reduce location-related effects. All procedures were conducted at the same time of day by the same investigators using standardized protocols, with each cage accommodating rats implanted with both materials. Blinding was ensured by having all samples evaluated by a pathologist and a micro-CT examiner who were blinded to the experimental protocol.

The biocompatibility of the Mg–Nd and Mg–Zn materials was assessed using two in vivo experimental models: subcutaneous implantation and bone implantation.

The sample size was determined using G*Power 3.1 software. The significance level was set at 0.05 and the statistical power at 0.80. Based on an effect size of Cohen’s (derived from reference data), a minimum of 4 animals per group was required. This method was selected to achieve an appropriate balance between statistical robustness and ethical considerations in the absence of reliable prior estimates of effect size.

Therefore, the subcutaneous implantation study included 32 rats, with euthanasia performed at four predefined time points (1, 2, 4, and 8 weeks post-implantation). The bone implantation study comprised 40 rats, with euthanasia conducted at the same corresponding time points for each experimental group. The experimental protocol was designed in accordance with the PREPARE guidelines and complied with Directive 2010/63/EU. In accordance with Romanian National Law no. 43/2014, the study was approved by the Institutional Ethics Committee (Decision no. 386/15.06.2023) and authorized by the Cluj Sanitary-Veterinary and Food Safety Department (Authorization no. 382/25.08.2023). All procedures were carried out in strict compliance with the approved protocols by highly trained personnel, applying well-established humane endpoint criteria (based on pain intensity RGS method, body weight loss, food and water consumption, and body condition scoring), with moderate severity defined as the highest acceptable cumulative level.

#### 2.3.1. Sterilization of Biomaterials

All magnesium-based powder specimens were sterilized under aseptic conditions using ultraviolet (UV-C) irradiation (254 nm, 30 min) in a laminar flow biosafety cabinet. UV-C was selected as a non-thermal, moisture-free method to avoid initiating corrosion of the magnesium alloys, which can occur when exposed to pressurized steam during autoclaving. Such exposure may promote the formation of magnesium hydroxide, release hydrogen gas, and alter the surface integrity and mechanical properties of the implants. Following sterilization, samples were handled exclusively with sterile instruments and stored in sealed sterile containers until implantation.

#### 2.3.2. Anesthesia Protocol

Anesthesia was induced via intraperitoneal injection using 80 mg/kg ketamine (Narkamon Bio 10%, Bioveta, Ivanovice na Hané, Czech Republic) and 0.5 mg/kg dexmedetomidine (Maravet, Cicarlau, Romania). Analgesia was achieved by using tramadol (KRKKA, Novo Mesto, Slovenia) 15 mg/kg intramuscular injection. Sterile ocular gel (Optixcare^®^, CLC medica, Ontario, Canada) was applied to avoid drying the cornea. The depth of the anesthesia was checked throughout the procedure with continuous monitoring to ensure the absence of the pedal withdrawal reflex and regular respiratory rates. A heating blanket (Therapeutic Prod., Beijing, China) was used for thermal support. Atipamezole (Narcostop^®^, Produlab Pharma B.V., Raamsdonksveer, The Netherlands) was used to reverse the effect of dexmedetomidine after the surgery.

#### 2.3.3. Surgical Protocol

**Figure 1:**
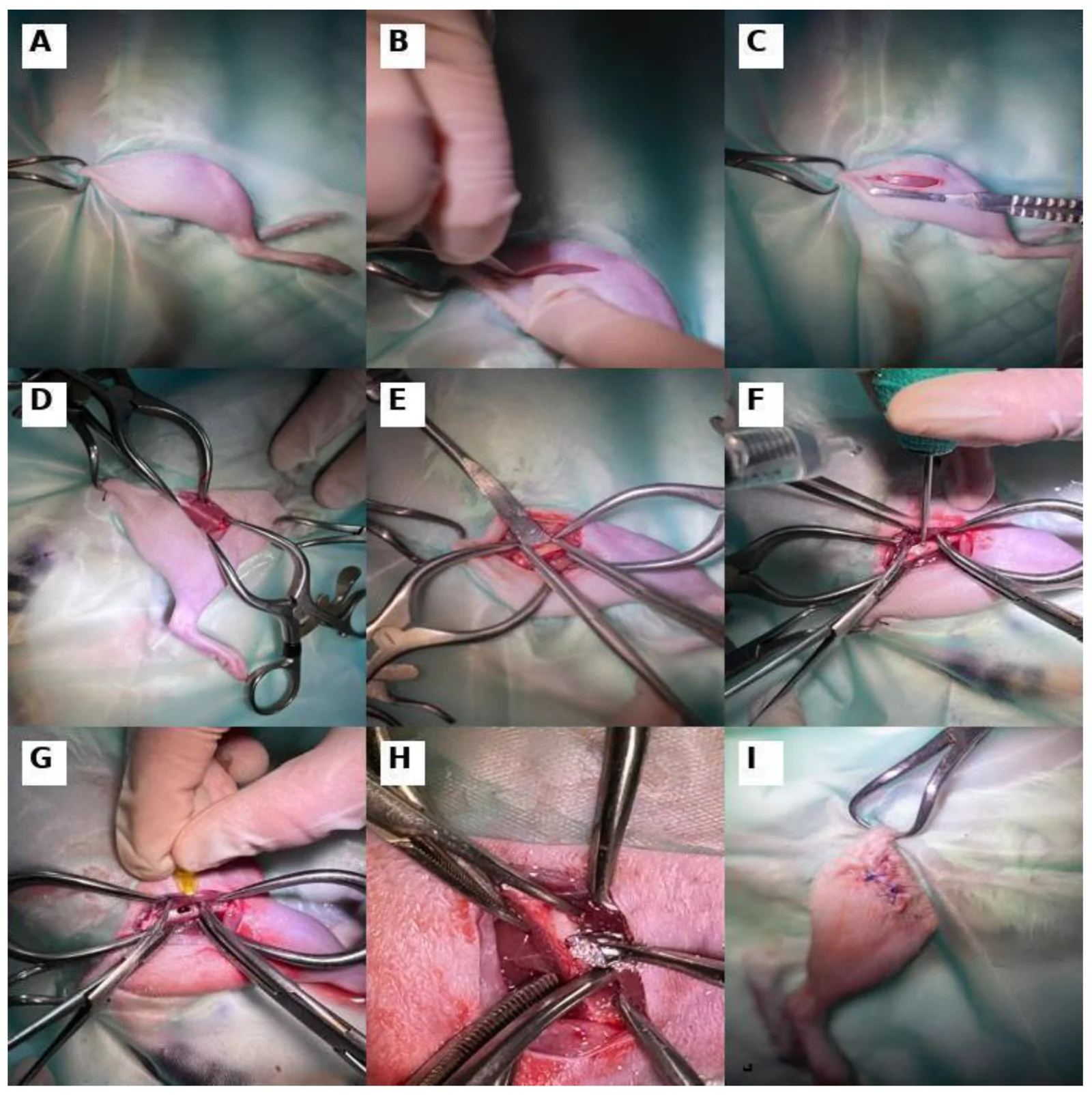
(**A**) Right pelvic limb prepared for surgery, (**B**) skin incision, (**C**) size of the skin incision, (**D**) aspect of the femoral diaphysis, (**E**) isolation of the femur, (**F**) induction of bone defect, (**G**) measurement of the defect, (**H**) filling the defect with powdered Mg alloys, (**I**) macroscopic aspect post-intervention.

The entire right hind limb and dorsal aspect was clipped and prepared for surgery using an electric shaver (Heiniger, Herzogenbuchsee, Switzerland). The rats were positioned in lateral recumbency on the surgical table (Figure 1). The surgical area was disinfected with 4% chlorhexidine (Lifo-Scrub^®^, B. Braun Melsungen AG, Melsungen, Germany) and then 70% alcohol (Alcool Sanitar 70%, Dual Prod, Satu Mare, Romania). A cranio-lateral skin incision, approximately 2.5 cm in length, was made along the long axis of the femur using an 11-blade disposable surgical scalpel. Following this, a meticulous incision was performed through the intersection of the biceps femoris and fascia lata muscles with Metzembaum scissors, carefully avoiding vascular injury by utilizing the intermuscular septum.

Blunt dissection of the muscle attached to the femur was performed with a periosteal elevator, exposing the middle of the femoral diaphysis while ensuring the periosteum remained intact. Gelpi retractors were deployed to improve the surgical field visibility. A needle bevel was used to mark the defect site on the femoral diaphysis. An oral low-speed handpiece, fitted with a 1.2 mm diameter diamond-tipped ball drill, was placed perpendicular to the bone surface at the marked site. This tool was used to create a box-shaped cavity defect, with continuous irrigation using 0.9% saline to prevent thermal necrosis of the bone tissue.

An oral low-speed handpiece, fitted with a 1.2 mm diameter diamond-tipped ball drill, was placed perpendicular to the bone surface at the marked site. This tool was used to create a cylindrical bone defect under continuous irrigation with 0.9% saline to prevent thermal necrosis of the tissue. For consistency, the length of the defect was controlled using a needle shaft marked at 0.3 mm, positioned parallel to the defect’s edge. Powdered magnesium alloys were then carefully deposited into the defect using a Volkmann spoon, ensuring complete coverage. The muscular fascia was sutured using 4-0 PDO monofilament suture material (AssuCryl^®^ MonoSlow, Assut Medical, Switzerland) in a continuous pattern. This was followed by subcutaneous suturing with the same material. The skin incision was closed using 4-0 non-resorbable nylon suture material (Ethilon^®^, Ethicon, Johnson & Johnson Medical Devices), employing a superficial interrupted “X” pattern to ensure proper wound apposition and healing.

#### 2.3.4. Subcutaneous Defect

**Figure 2:**
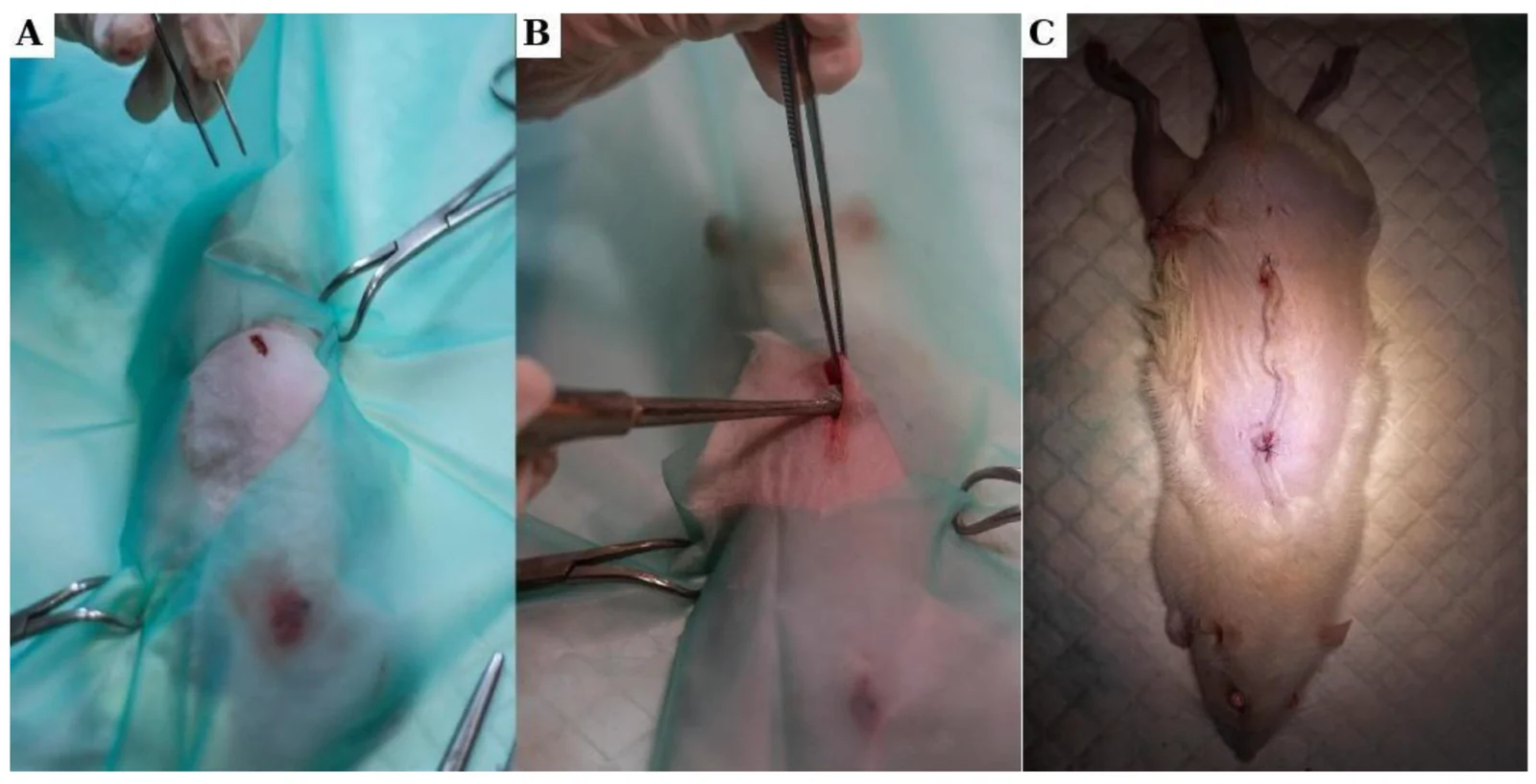
(**A**) Skin incision, (**B**) subcutaneous placing of powdered Mg alloy, (**C**) macroscopic aspect post-intervention.

The rats were positioned in sterno-abdominal recumbency for procedures involving subcutaneous defects (Figure 2). The skin was disinfected following the same protocol used for bone defects, involving 4% chlorhexidine followed by 70% alcohol. A small cranial incision was made using an 11-blade scalpel, through which 140 mg of powdered magnesium alloys were placed using a Volkmann spoon.

Similarly, a small caudal incision was made to insert the same biomaterial in rod form, measuring 0.3 cm in length, 0.2 cm in width, and 0.1 cm in height. The skin incisions were then closed using 4-0 non-resorbable nylon monofilament sutures, employing a superficial interrupted “X” pattern to ensure proper wound closure and healing.

#### 2.3.5. Postoperative Care

Minor complications, such as subcutaneous emphysema, were observed following surgery, due to corrosion of the biomaterial and hydrogen gas formation. After the procedure, the animals were allowed to move freely within their cages and were provided with fluids (NaCl 0.9%, B. Braun Melsungen AG, Melsungen, Germany) for 2–3 days postoperatively. Pain management included the administration of tramadol at a dose of 15 mg/kg intramuscularly and metamizole at a starting dose of 150 mg/kg orally, with the dose adjusted based on pain scores assessed using the rat grimace scale. 10 mg/kg of enrofloxacin (Enrofloxacină FP 10%, Farmavet, Chișinău, Republica Moldova) was administered every 24 h for 5 days for antibiotic prophylaxis. The skin wounds healed without complications within 7–10 days post-surgery.

Rats were euthanized at 1, 2, 4 and 8 weeks after the implantation using carbon dioxide exposure followed by occipito-atloidian luxation after cessation of respiratory and heart activity. Skin samples and femoral bone specimens were collected and fixed in 4% formaldehyde for subsequent histopathological examination and micro-CT scanning.

### 2.4. Ex Vivo Studies

#### 2.4.1. CT Scanning and Post-Processing Methods

**Figure 3:**
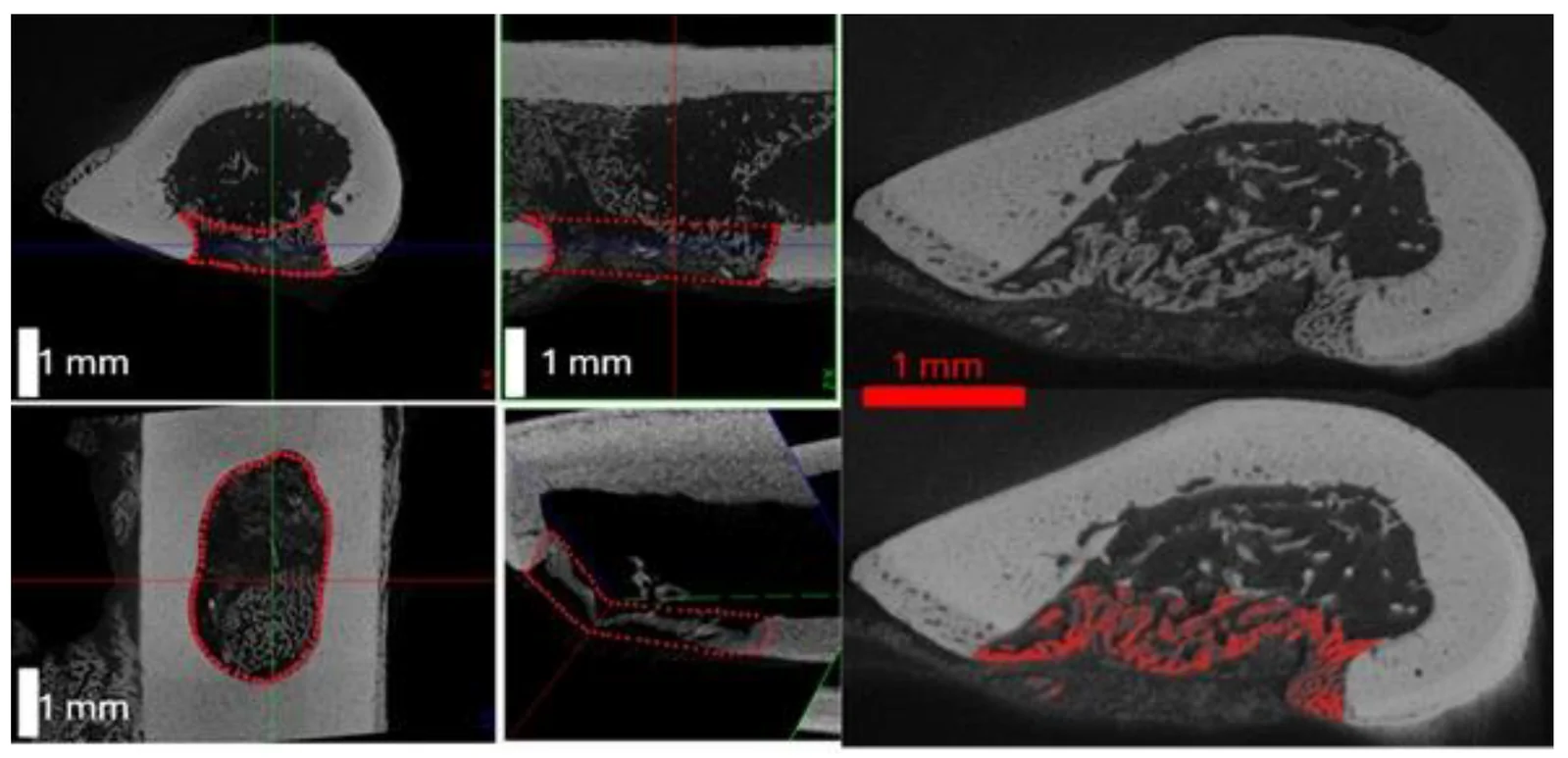
Exemplification of manual VOI definition in the three planes and plane intersection (red dots), as well as in a tomogram slice depicting healthy femur, medullary channel, mineralization within the medullary channel, mineralization within the defect, soft tissue surrounding the neoformation bone; in red, the BV in the respective slice considered for analysis.

Bone specimens were scanned using a Bruker Skyscan 1272 micro-computed tomography system (Bruker microCT, Kontich, Belgium). Scanning was conducted under the following parameters: an X-ray tube voltage of 70 kV and a current of 140 μA. An exposure time of 1400 ms per projection was used with a frame averaging of 3 to enhance signal-to-noise ratio. The specimens were rotated over 360°, with a rotation step of 0.2° between projections. A 0.5 mm aluminum filter was applied to reduce beam hardening artifacts. Projection images were acquired at a detector resolution of 2452 × 1640 pixels, resulting in a reconstructed voxel size of 3.5 μm [32,33]. Reconstruction of raw data was performed using the manufacturer’s software (NRecon, 1.7.1.6 software, Bruker, Billerica, MA, USA), incorporating correction for beam hardening and ring artifacts where necessary. Subsequent quantitative analysis of bone morphometry was carried out using CTAn (Bruker, Billerica, MA, USA), with a consistent volume of interest (VOI) defined across samples (Figure 3). To assess bone formation within the defect region, a VOI was manually delineated using CTAn software. The defect was an oval-shaped cortical window surgically created in the mid-diaphysis of the femur, where the biomaterial was implanted. Given the potential migration of the biomaterial and bone formation extending into the medullary canal, only the originally intended defect region was considered for analysis.

The VOI was manually defined slice by slice, following the boundaries of the adjacent cortical bone. To approximate the original extent of the defect, the VOI limits were determined by visually reconstructing the expected continuity of the cortical bone. Although the surgical incision initially created a defined window, the observed defect margins appeared irregular, likely due to bone remodeling during the implantation period. This delineation was performed to ensure that only bone formation within the intended defect area was included, while excluding regions where the biomaterial had migrated or where bone formation occurred outside the defect. Variations in VOI size were influenced by differences in cortical bone thickness delimiting the surgical window, as well as by bone resorption that may have occurred during the implantation period.

The VOI consists of bone volume (BV) and surrounding soft tissue structures. Upon applying a threshold, BV was represented by white pixels, while the remaining tissue was depicted in black pixels, which was subsequently referred to as porosity.

##### Bone Mineral Density

Bone mineral density (BMD) values were obtained from ex vivo micro-CT scans of explanted samples at five time points: 1, 2, 4, and 8 weeks post-implantation. BMD was reported in units of g/cm^3^, calculated (CTAn 1.17.7.2 software) from calibrated CT attenuation coefficient values using hydroxyapatite phantoms as reference standards.

#### 2.4.2. Histopathology

Collected tissues were fixed in 10% formalin for 48 h, dehydrated through graded ethanol, cleared in xylene, and embedded in low-melting-point paraffin at 58 °C for 5 h. Sections (2 µm) were cut using a rotary microtome. Slides were deparaffinized in xylene, rehydrated through graded ethanol, and rinsed in tap water. Sections were stained with hematoxylin for 5 min and eosin for 2 min, followed by dehydration in ethanol, clearing in xylene, and mounting with Permount. For bone samples, after fixation, specimens were decalcified in a rapid decalcifying solution for 24 h and subsequently transferred to 70% ethanol prior to routine dehydration and paraffin embedding. Histological examination was performed using an Olympus BX51 light microscope under bright-field illumination. Images were captured with an Olympus SP350 digital camera (Olympus Corporation, Tokyo, Japan) and processed using Olympus cellSens™ imaging software version 3.1. Microscopic tissue changes were evaluated and graded for severity in accordance with ISO/DIS 10993-6:2024 (en).

#### 2.4.3. Statistical Analysis

Data are presented as mean standard deviation (SD). Normality of the datasets was assessed using the Shapiro–Wilk test. As all data satisfied the assumption of normality, a one-way ANOVA was employed to analyze the cell proliferation assay. Two-way ANOVA followed by Tukey’s post hoc multiple comparisons test was applied for micro-CT structural parameters, including BMD, bone surface (BS), BS/BV, bone volume (BV), bone volume fraction (BV/TV), and total volume (TV).

Statistical significance was defined as *p* < 0.05 (95% confidence interval). Statistical analyses and graphical representations were performed using GraphPad Prism^®^ version 10 for Windows, GraphPad Software, San Diego, CA, USA.

## 3. Results

### 3.1. Characterization of Experimental Samples by Microscopic Techniques and X-Ray Diffraction

#### 3.1.1. Optical Microscopy

**Figure 4:**
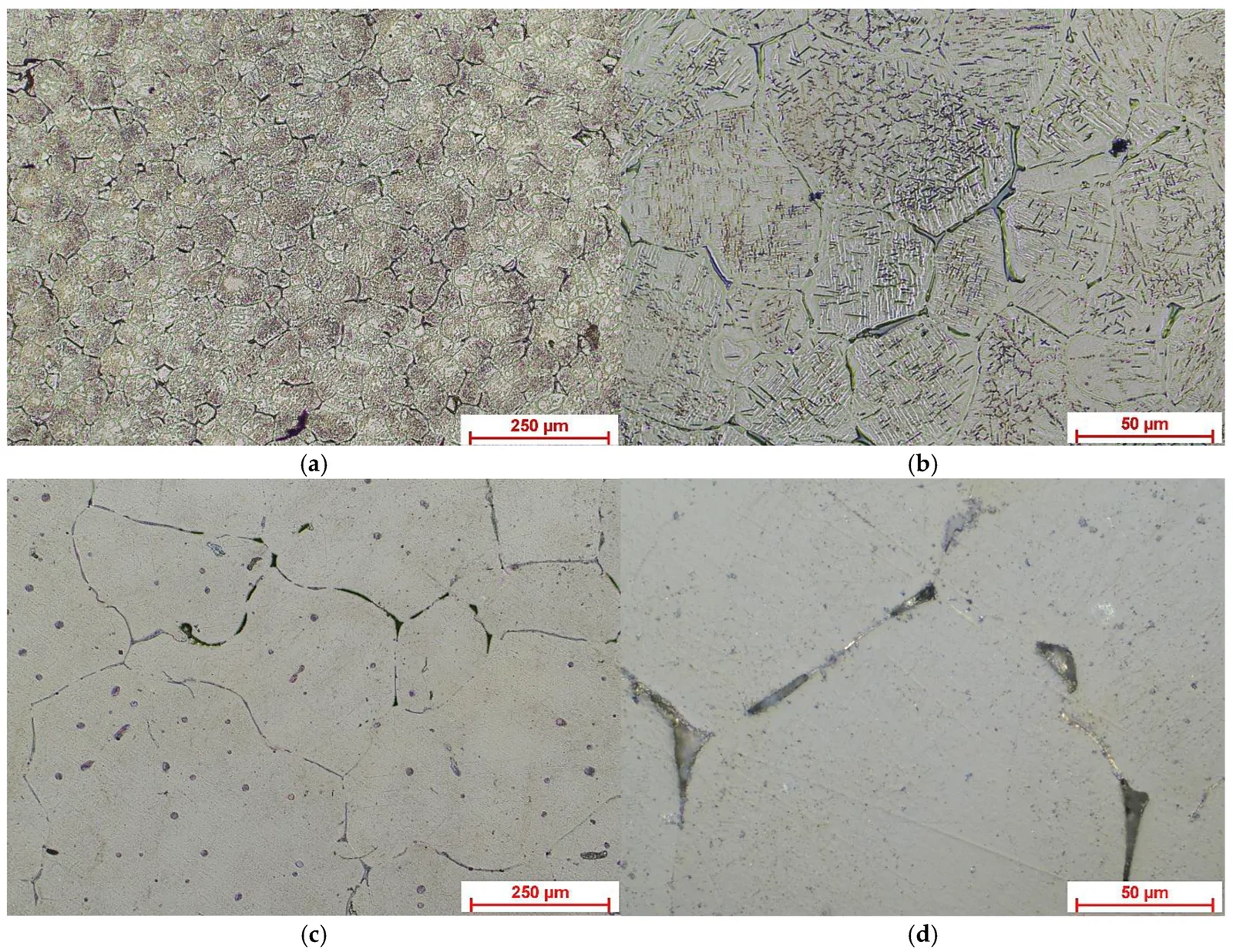
Optical microscopy images of the investigated alloys at different magnifications: (**a**) MRI 201s, magnification 10X; (**b**) MRI 201s, magnification 50X; (**c**) ZMX 410, magnification 10X; (**d**) ZMX 410, magnification 50X.

The first alloy incorporates zinc (Zn) along with two rare earth elements, yttrium (Y) and neodymium (Nd), as alloying constituents. In contrast, the investigated ZMX 410 alloy includes additional alloying elements, an alkaline earth metal, calcium (Ca), and a transition metal, manganese (Mn). Figure 4 presents the microstructural analysis of MRI 201s and ZMX 410 alloys, which reveals notable differences in grain morphology, phase distribution, and overall structural uniformity.

The optical micrographs of the MRI 201s alloy exhibit a uniform distribution of polyhedral α-Mg grains, with secondary phases present within the grains in both acicular and globular morphologies, as well as at the grain boundaries, which are likely of a multicomponent nature. In contrast, the ZMX 410 alloy presents larger and less uniform α-Mg grains, along with intragranular secondary phase precipitates and a eutectic phase located at the grain boundaries. The MRI 201s alloy shows a higher degree of intragranular precipitation and the presence of acicular phases compared to ZMX 410. However, the MRI 201s alloy exhibited a more refined and homogeneous microstructure overall, compared with ZMX 410.

#### 3.1.2. X-Ray Diffraction Analysis Results (XRD)

**Figure 5:**
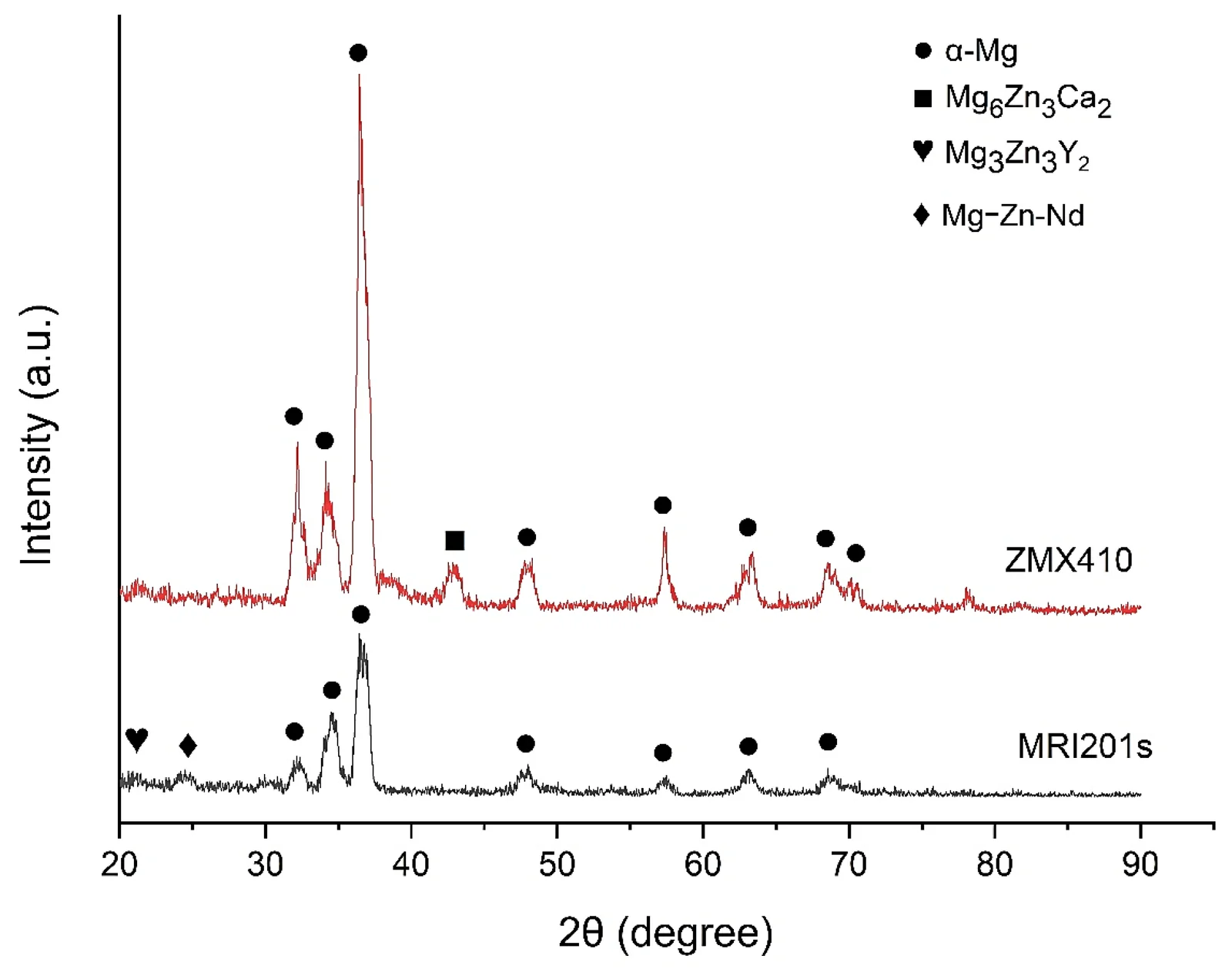
X-ray diffraction patterns of the MRI 201s and ZMX 410 alloys.

The XRD patterns of the investigated magnesium alloys are shown in Figure 5. For the MRI 201s alloy, diffraction peaks corresponding to the α-Mg phase, Mg–Zn–Nd phases, and the W-phase (Mg_3_Zn_3_Y_2_) were identified. No diffraction peaks attributable to the I-phase (Mg_3_Zn_6_Y) were detected. For the ZMX 410 alloy, the XRD pattern showed diffraction peaks corresponding to the α-Mg and Mg_6_Zn_3_Ca_2_ phases. No diffraction peaks attributable to Mg_2_Ca or MgZn phases were identified under the applied experimental conditions.

#### 3.1.3. Scanning Electron Microscopy Coupled with Energy Dispersive Spectroscopy (SEM-EDS)

**Figure 6:**
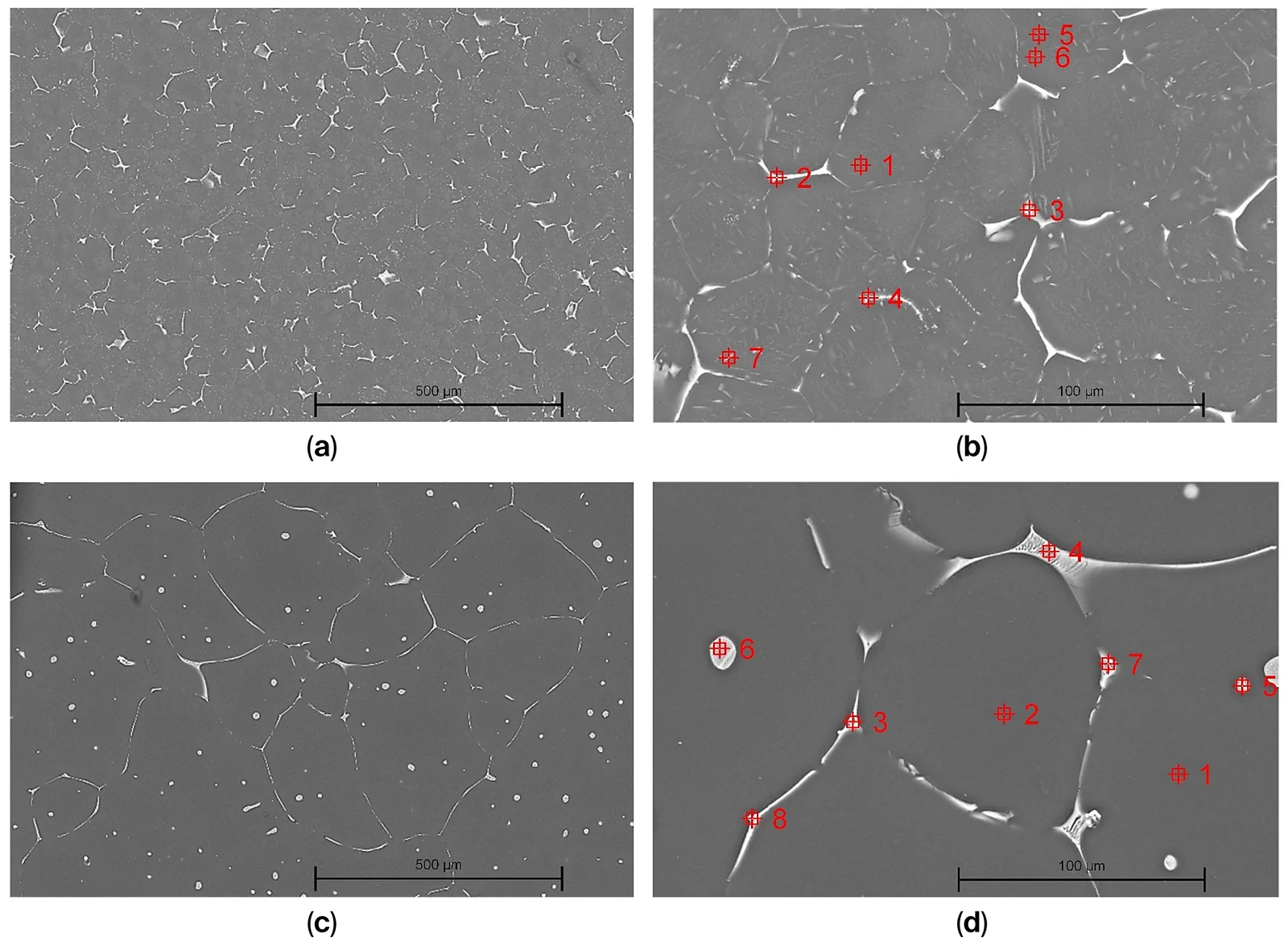
SEM images of the alloys investigated at different magnifications: (**a**) MRI 201s, magnification 100X; (**b**) MRI 201s, magnification 500X; (**c**) ZMX 410, magnification 100X; (**d**) ZMX 410, magnification 500X.

**Figure 7:**
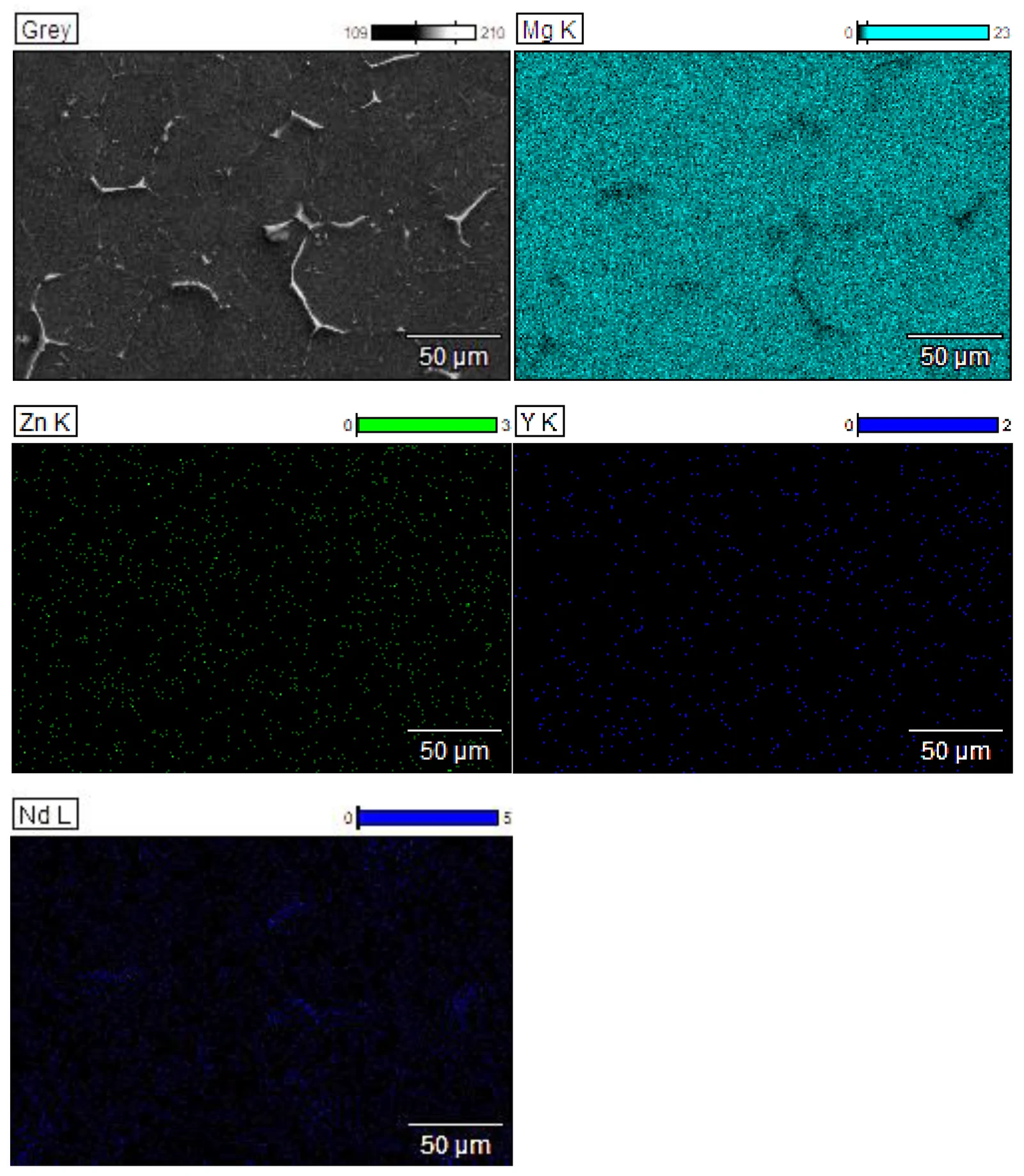
Results of the EDS analysis performed on the MRI 201s alloy.

**Figure 8:**
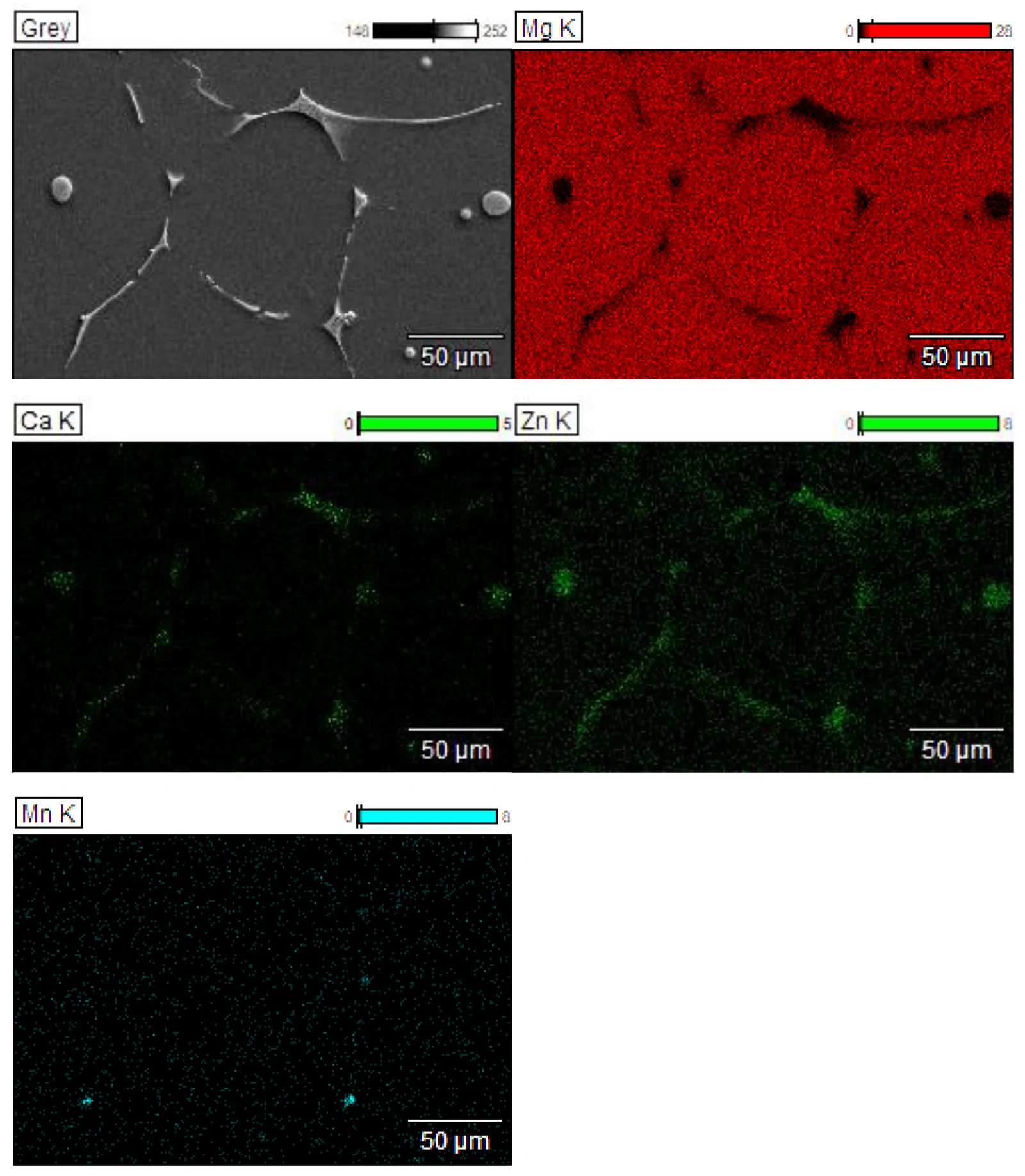
Results of the EDS analysis performed on the ZMX 410 alloy.

SEM analysis of the MRI 201s and ZMX 410 alloys revealed α-Mg grains of different sizes, together with secondary phases located both within the grains and at the grain boundaries (Figure 6). In the MRI 201s alloy, intragranular secondary phases exhibited globular and acicular morphologies. EDS elemental mapping detected Mg, Zn, Y, and Nd in the analyzed regions of the α-Mg matrix and grain boundaries (Figure 7, Table 2). Points 2–4 showed increased concentrations of Nd, Y, and Zn compared with the α-Mg matrix, whereas Mg, Zn, and Nd were identified in the acicular and globular secondary phases corresponding to points 5–7. SEM–EDS analysis of the ZMX 410 alloy revealed Zn and Mn within the α-Mg grains (Figure 8, Table 2, points 1–2). A secondary phase containing Mg, Ca, and Zn was detected both at the grain boundaries and within the grains. Points 3–6 showed Zn concentrations ranging from approximately 25 to 43 wt% and Ca concentrations ranging from 5.5 to 9.4 wt%, with no detectable Mn. Points 7 and 8 showed Mn concentrations of approximately 31 wt% and 45 wt%, respectively, together with lower Zn and Ca concentrations. Based on the combined SEM–EDS and XRD results, α-Mg and Mg_6_Zn_3_Ca_2_ phases were identified in the ZMX 410 alloy, together with Mn-rich regions detected by EDS.

### 3.2. Cytotoxicity Testing

**Figure 9:**
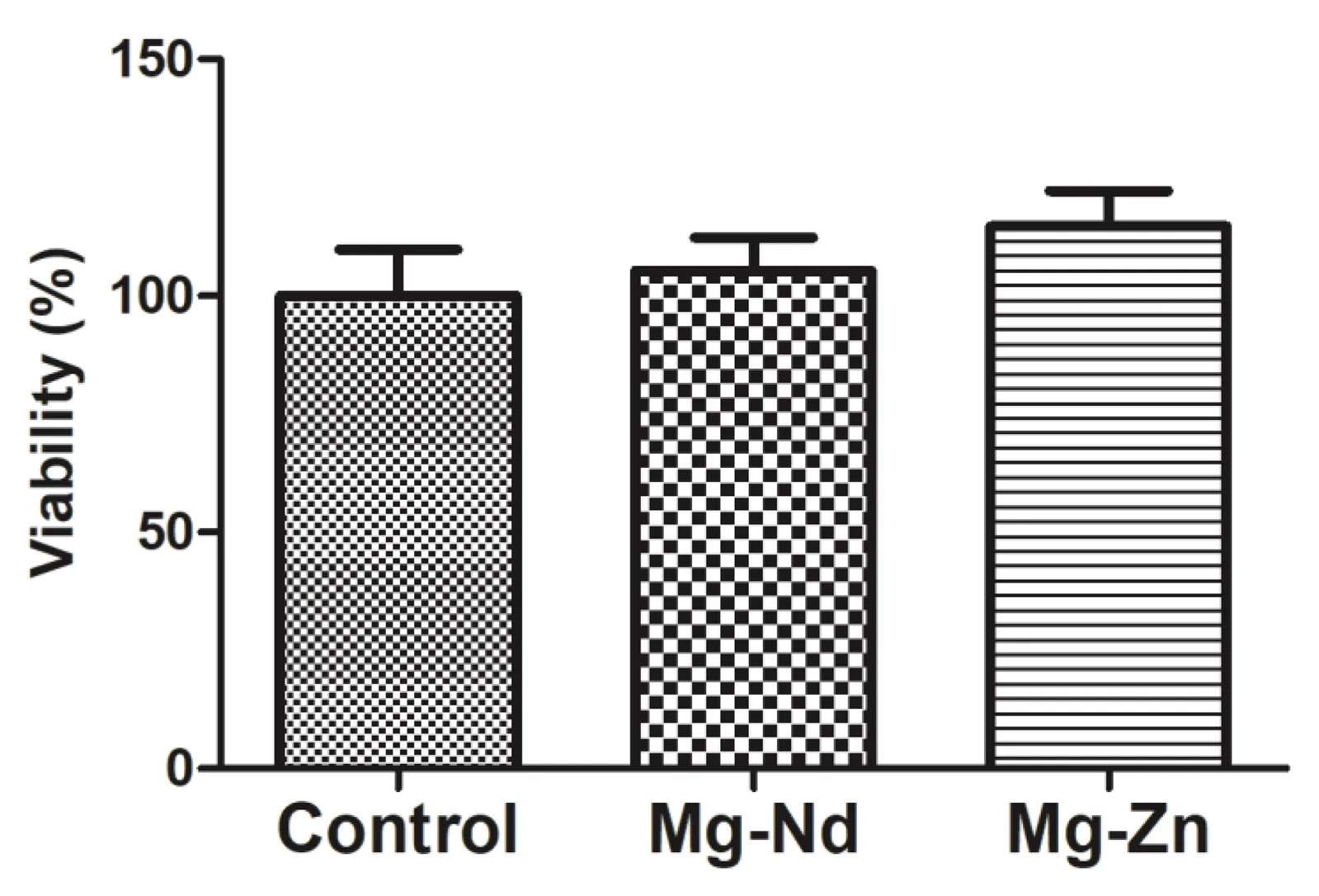
Viability of osteoblast cells after 24 h interaction with MRI 201s (Mg-Nd) and ZMX 410 (Mg-Zn) (mean ± SEM; *n* = 3).

Alloys after 24 h, cellular viability was between 97% and 112% for MRI 201s and between 101% and 115% for ZMX 410, respectively, therefore both alloys had no cytotoxic effect on the osteoblast cell culture (Figure 9). Moreover, the samples had slightly proliferative effect on the osteoblast cells, more visible for the ZMX 410 alloy; however, the difference was not statistically significant.

### 3.3. Microbial Minimum Inhibitory Concentration

The MIC values obtained for MRI 201s and ZMX 410 against the tested bacterial and fungal strains are presented in Table 3. For Pseudomonas aeruginosa ATCC^®^ 27853, MIC values were 2.88 mg dry weight/mL for MRI 201s and 2.96 mg dry weight/mL for ZMX 410. For Staphylococcus aureus subsp. aureus ATCC^®^ 29213, an MIC of 46.07 mg dry weight/mL was recorded for MRI 201s, whereas no bioactivity was detected for ZMX 410 at the tested concentrations. For Salmonella enterica ATCC 14028, MIC values were 46.07 mg dry weight/mL for MRI 201s and 47.50 mg dry weight/mL for ZMX 410.

For Candida albicans DSMZ 1386, MIC values were 11.51 mg dry weight/mL for MRI 201s and 11.87 mg dry weight/mL for ZMX 410. No bioactivity was detected against Candida parapsilosis ATCC^®^ 22019 for either alloy at the tested concentrations. For Staphylococcus epidermidis ATCC^®^ 12228, an MIC of 47.50 mg dry weight/mL was recorded for ZMX 410, whereas no bioactivity was detected for MRI 201s at the tested concentrations.

### 3.4. Micro-CT Assessment

**Figure 10:**
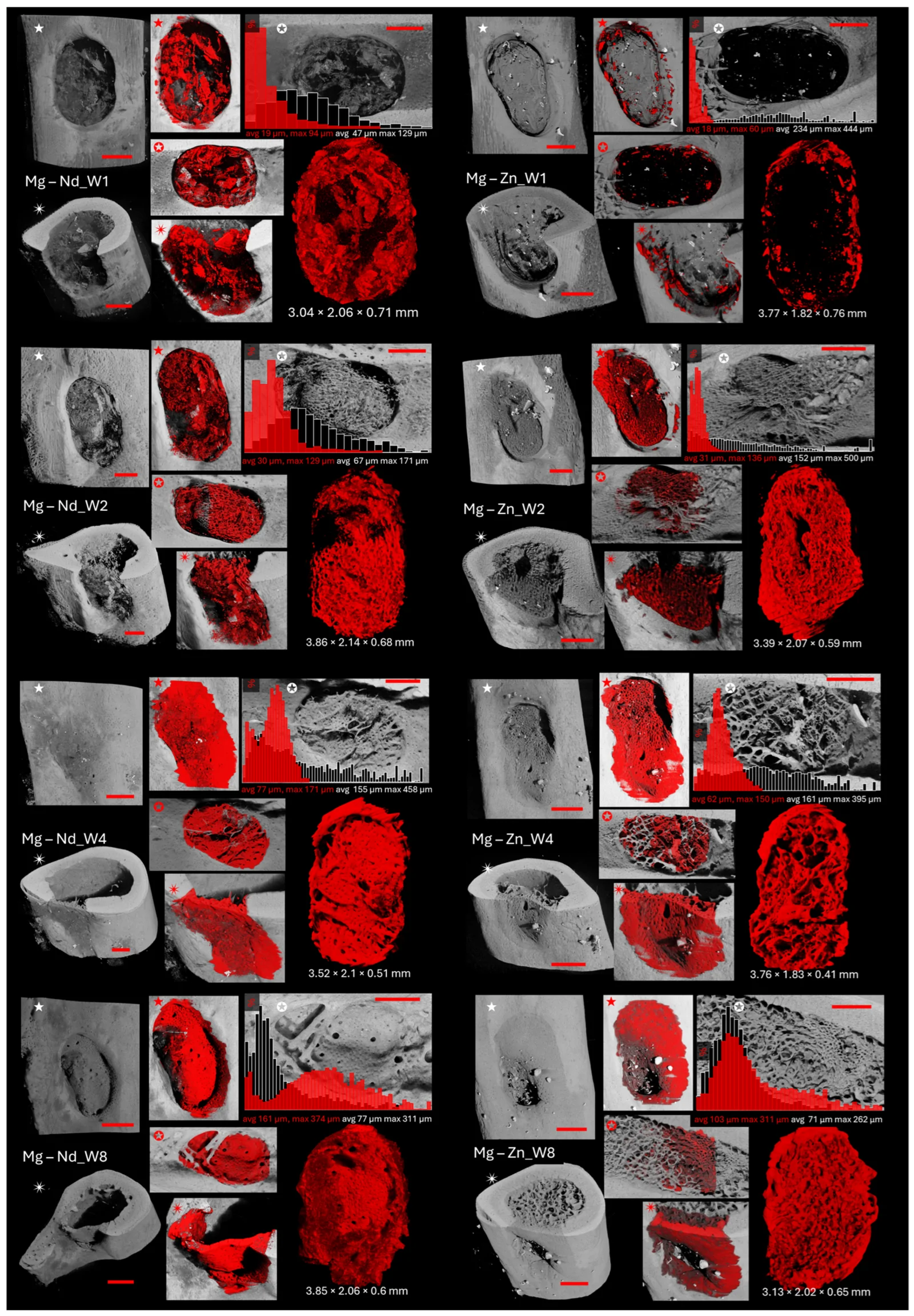
Micro CT images of Mg-Nd and Mg-Zn explants after 1, 2, 4 and 8 weeks of implantation illustrating (★) frontal view of the bone defect with BV formed within; (✪) the view of the BV from inside the medullary channel; (✴) cross-sectional view of the femur (mid-incision window level), with the contrast adjusted to focus on the mineral phase. Inset histograms (red—BV; black—pore/marrow space) illustrate the size distribution of the two phases over time, with mean and maximum equivalent diameter reported below each chart. Red-colored tomogram solely illustrates the BV with maximum dimensions of the volume of interest reported as L × l × w (mm). Red scale bar: 1 mm.

During the evolution of bone morphology over time, the structural characteristics of the femoral bone defect changed progressively across the evaluated time points (Figure 10). In the early stage, particularly at Week 1, the defect region exhibited limited mineralized tissue and only a few trabecular structures. The incision window showed disparate centers of mineralization in both the MRI 201s and ZMX 410 groups.

By Week 2, an increase in mineralized tissue was observed, together with the appearance of an initial trabecular structure. The newly formed mineralized tissue displayed an irregular architecture, and the first trabecular bridges became visible. By Week 4, a more organized trabecular arrangement was observed, together with an increase in mineralized tissue and a more continuous interface with the adjacent native bone. Non-mineralized regions within the VOIs became less prominent, while the mineralized domains appeared more coalescent and formed a more continuous structure across the defect.

By Week 8, the defect region showed a more continuous and organized mineralized network, with increased trabecular interconnectivity compared with the earlier time points. The newly formed mineralized tissue exhibited fewer open regions and thicker mineralized structures, particularly at the external surface of the femur. Residual differences in architecture remained visible within the medullary region, where a higher proportion of trabecular structures was still present.

**Figure 11:**
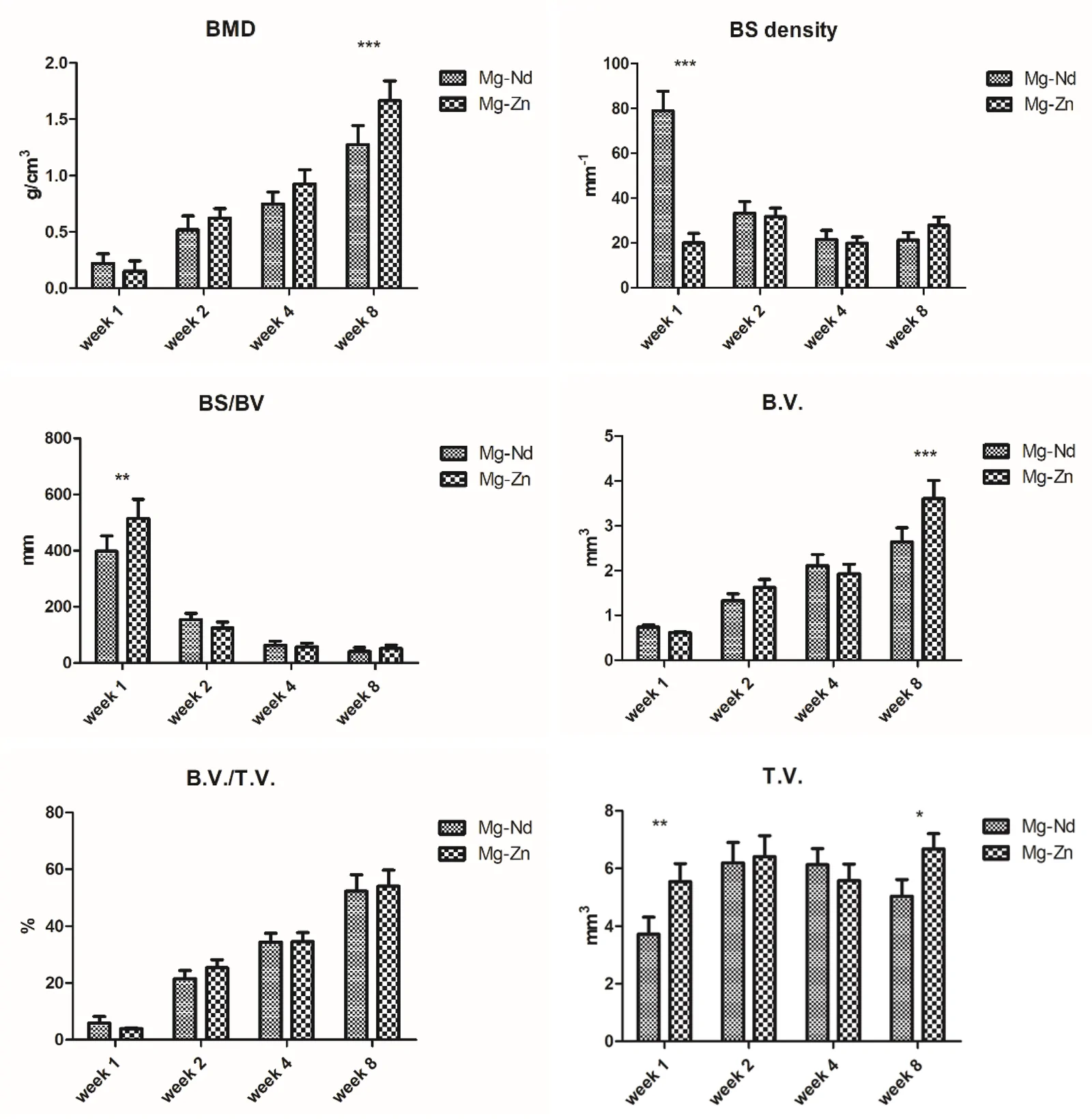
Bone mineral density (BMD) and bone specific surface (BS/BV) at different time points. (mean ± SD, *n* = 4, two-way ANOVA, Tuckey post-test, * *p* < 0.05; ** *p* < 0.01; *** *p* < 0.001).

To characterize the structural changes within the defect region, several microarchitectural parameters were assessed at the different explantation time points. Bone volume (BV) was evaluated as the absolute volume of mineralized tissue (mm^3^), its proportion within the VOI, surface density, surface-to-volume ratio, and thickness-related parameters. Both materials showed an increase in BV and the percentage of bone within the VOI over the evaluated time points. ZMX 410 showed lower bone formation at Week 1, whereas higher bone volume values were recorded at Week 8. MRI 201s showed high surface-density values at the early time points, which decreased as bone volume increased. Differences between the two materials were also observed in the surface-to-volume parameters over time, with lower surface per unit volume recorded for ZMX 410 from Week 2 onward. Residual differences in microarchitecture, including porosity, trabecular organization, and mineral density, were still observed at Week 8. Changes in these parameters over time are presented in Figure 10 and Figure 11.

### 3.5. Histopathological Examination

#### 3.5.1. Subcutaneous Implantation

**Figure 12:**
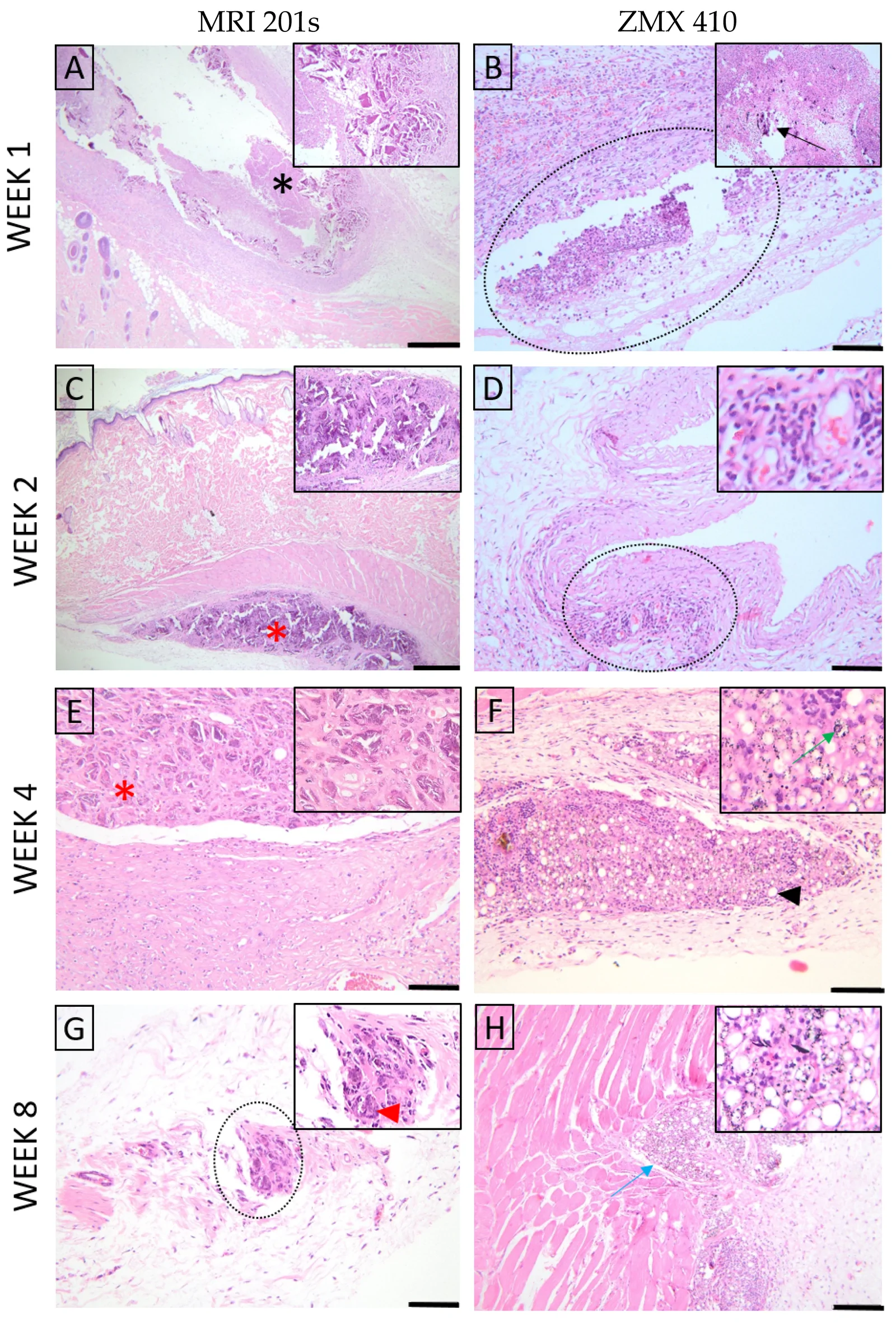
Histopathological evaluation of skin and subcutis lesions. (Image (**A**)) Skin and subcutis displaying moderate necrosis (black asterisk) and severe infiltration by polymorphonuclear cells, with minimal lymphocytes, mild macrophages, and moderate multinucleated giant cells. Severe neovascularisation and mild fibrosis are present. Inset: higher magnification of the inflammatory infiltrate. (Image (**B**)) Severe necrosis (dotted ellipse) associated with severe polymorphonuclear cell infiltration, mild macrophages, minimal giant cells. Inset: area of necrosis with neutrophilic debris (black arrow). (Image (**C**)) Minimal lymphocyte infiltration with moderate macrophages and mild multinucleated giant cells. A necrotic area admixed with giant cells is indicated (red asterisk). Inset: higher magnification of mixed inflammatory infiltrate. (Image (**D**)) Mild lymphocyte infiltration with severe macrophages and moderate giant cells within the lesion (dotted ellipse). Inset: higher magnification showing dense mononuclear infiltrate. (Image (**E**)) Minimal polymorphonuclear cells (red asterisk) and lymphocytes with moderate macrophages and minimal infiltration with giant cells. Inset: detail of macrophage-rich infiltrate. (Image (**F**)) Mild lymphocytes and plasma cells, moderate macrophages, and minimal giant cells are observed. The lesion shows abundant lipid-laden macrophages (black arrowhead). Inset: higher magnification highlighting intracytoplasmic lipid vacuoles admixed with a black-brown pigment (green arrow). (Image (**G**)) Minimal polymorphonuclear cells and macrophages within a small lesion (dotted ellipse). Inset: focus of inflammatory infiltrate (red arrowhead). (Image (**H**)) Mild lymphocytes and severe macrophages infiltrating the muscle (blue arrow), with minimal giant cells infiltration. Inset: lipid-laden macrophages within the muscle tissue admixed with a black-brown pigment. H&E staining; Ob. ×4 (Images (**A**,**C**)), Ob. ×10 (Images (**B**,**D**,**G**,**H**)), Ob. ×20 (Images (**E**,**F**)); Barr 100 μm (Images (**E**,**F**)), 200 μm (Images (**B**,**D**,**G**,**H**)), 500 μm (Images (**A**,**C**)).

In subcutaneous implantation (Table 4, Figure 12), histopathological evaluation revealed that in the MRI 201s group, the total lesion score was highest at Week 1 (20.25), corresponding to a marked acute inflammatory response characterized by necrosis and intense polymorphonuclear cell infiltration, as illustrated in Figure 12A,B. A decrease was observed by Week 2 (8.75), followed by a score of 10.75 at Week 4, mainly associated with macrophage infiltration and neovascularization (Figure 12C–E). By Week 8, the total lesion score declined to 4, with minimal residual inflammation and no necrosis observed (Figure 12G).

In contrast, the ZMX 410 group showed a high initial lesion score at Week 1 (16.75), with acute inflammatory features, followed by a reduction at Week 2 (9). The total lesion score was 10.75 at Week 4 and increased to 12.75 at Week 8. At Week 8, the inflammatory infiltrate included lymphocytes, plasma cells, and macrophages, together with fatty infiltration and lipid-laden macrophages (Figure 12F,H).

Across the evaluated time points, the MRI 201s group showed a decrease in the total lesion score from 20.25 at Week 1 to 4 at Week 8, whereas the ZMX 410 group showed a decrease from 16.75 at Week 1 to 9 at Week 2, followed by scores of 10.75 at Week 4 and 12.75 at Week 8.

#### 3.5.2. Bone Implantation

**Figure 13:**
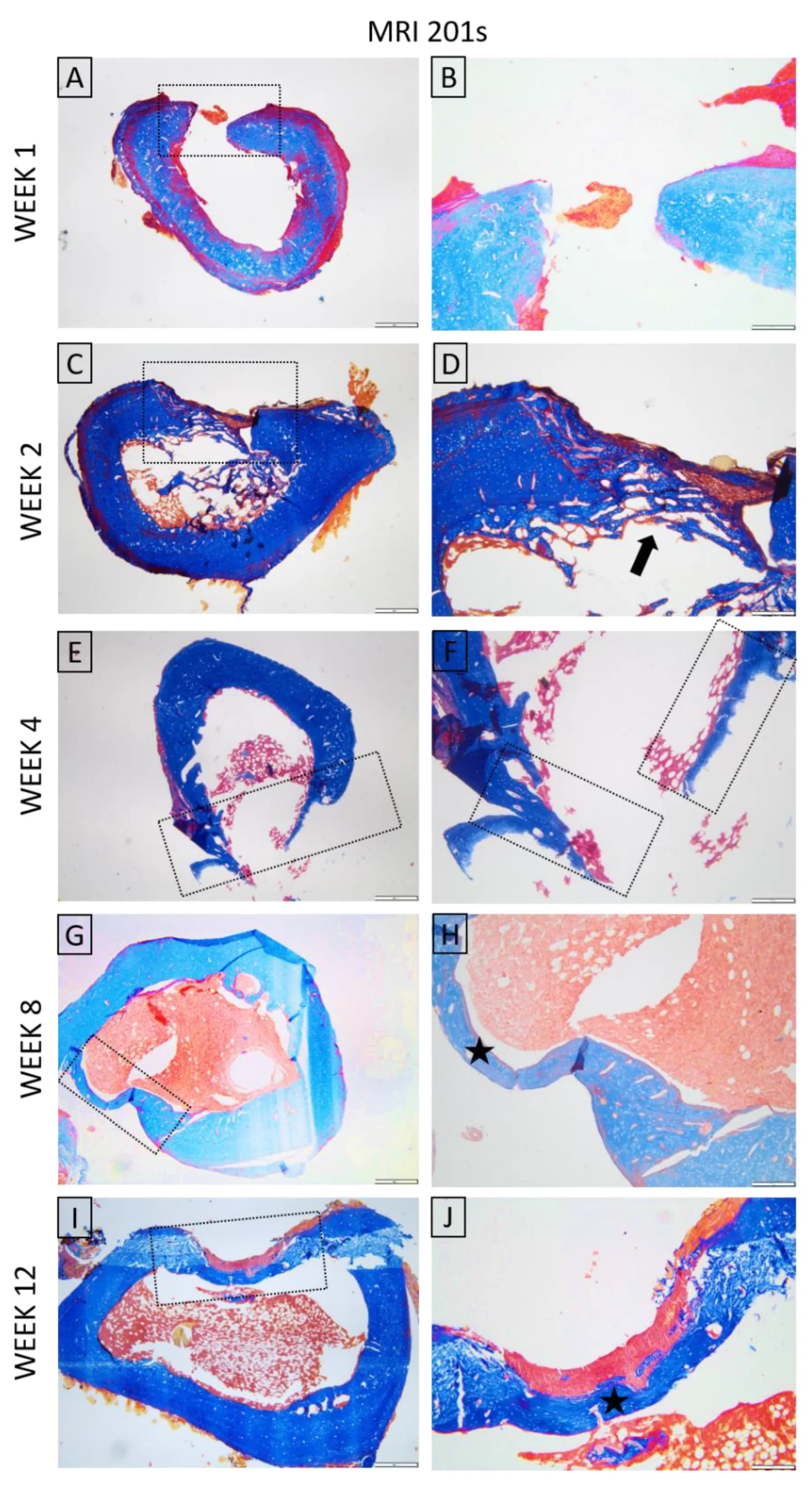
Histological aspect of the MRI 201s group. Long bone. Week 1 (Images (**A**,**B**)): The defect is present, and no signs of inflammation or tissue regeneration are observed, consistent with the early post-injury. Week 2 (Images (**C**,**D**)): Proliferation of endosteal and periosteal tissue minimally covers the defect, without inflammation (arrow). Week 4 (Images (**E**,**F**)): Defect completely covered by trabecular-like bone tissue, with minimal mononuclear inflammation; moderate periosteal and endosteal proliferation is observed around the defect. Week 8 (Images (**G**,**H**)): Proliferation of endosteal and periosteal tissue largely covering the defect, without inflammation (star). Week 12 (Images (**I**,**J**)): Defect completely covered by bone tissue with a compact lamellar appearance (star). Masson’s trichrome stain. Ob. ×4 (Images (**A**,**C**,**E**,**G**,**I**)) Ob. 10× (**B**,**D**,**F**,**H**,**J**). Scale bar 500 µm (Images (**A**,**C**,**E**,**G**,**I**)) and 200 µm (**B**,**D**,**F**,**H**,**J**).

**Figure 14:**
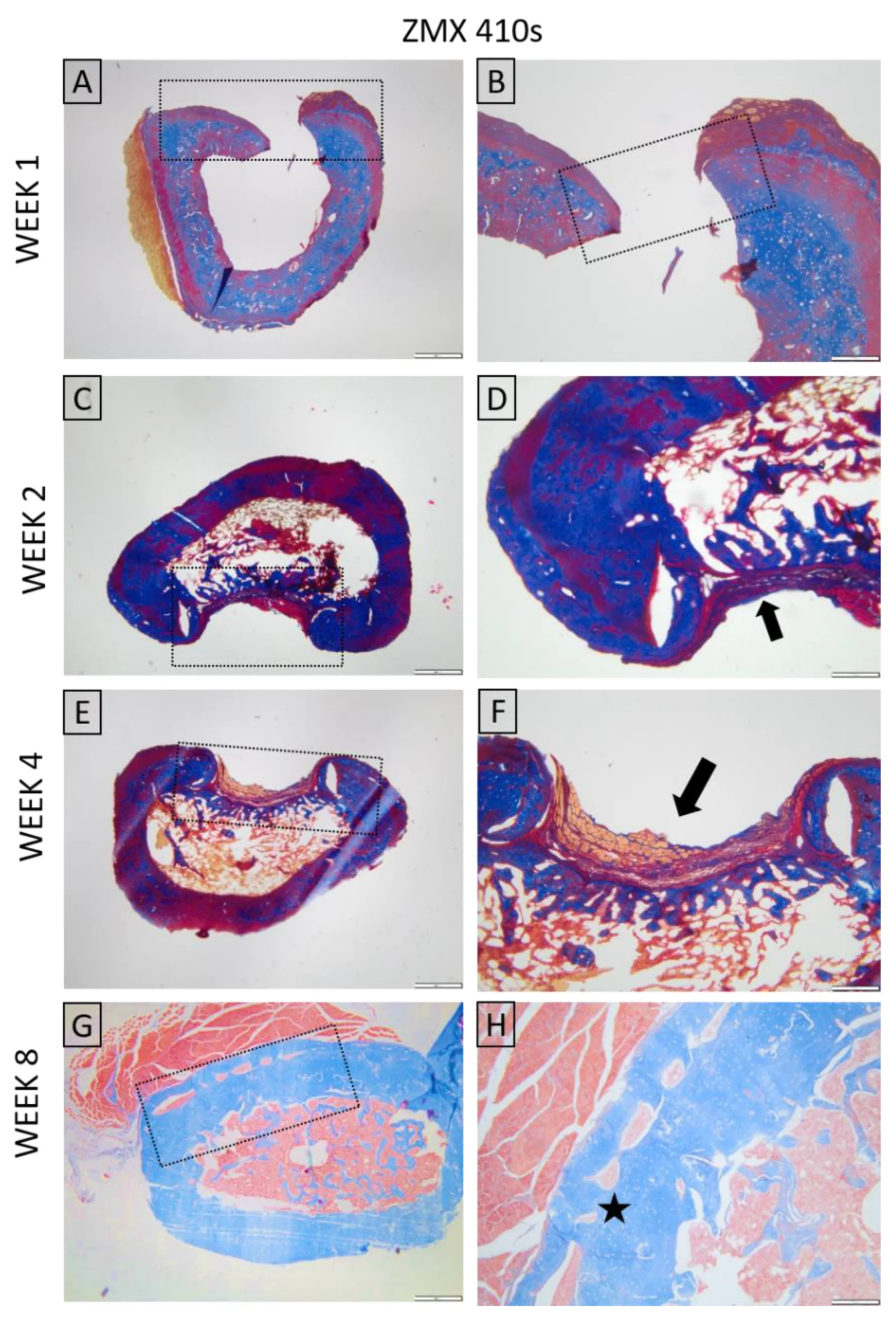
Histological aspect of the ZMX 410 group. Long bone. Week 1 (Images (**A**,**B**)): The defect is present, and no signs of inflammation or tissue regeneration are observed, consistent with the early post-injury. Week 2 (Images (**C**,**D**)): Proliferation of endosteal and periosteal tissue minimally covers the defect, without inflammation (arrow). Week 4 (Images (**E**,**F**)): Bone defect incompletely covered by bone trabecula (arrow). Week 8 (Images (**G**,**H**)): Defect completely covered by bone tissue with a compact lamellar appearance (star). Masson’s trichrome stain. Ob. ×4 (Images (**A**,**C**,**E**,**G**)) Ob. 10× (**B**,**D**,**F**,**H**). Scale bar 500 µm (Images (**A**,**C**,**E**,**G**)) and 200 µm (**B**,**D**,**F**,**H**).

Histological evaluation of bone defect healing (Figure 13 and Figure 14) showed time-dependent morphological changes in both experimental groups. In the ZMX 410 group, the defect was clearly identifiable at Week 1, without signs of inflammation or newly formed bone (Figure 13A,B). By Week 2, limited endosteal and periosteal proliferation was observed, with minimal coverage of the defect and absence of inflammatory infiltrate (Figure 13C,D). At Week 4, the defect remained incompletely filled, with immature trabecular bone formation (Figure 13E,F). By Week 8, complete defect closure was observed, with compact lamellar bone present within the defect region (Figure 13G,H).

In the MRI 201s group, a similar initial aspect was observed at Week 1, with a clearly defined defect and no evidence of inflammation or newly formed bone (Figure 14A,B). By Week 2, endosteal and periosteal proliferation was observed, contributing to partial coverage of the defect (Figure 14C,D). At Week 4, the defect was completely bridged by trabecular-like bone, accompanied by minimal mononuclear inflammatory infiltrate and moderate periosteal and endosteal proliferation (Figure 14E,F). At Week 8, extensive endosteal and periosteal proliferation was observed. By Week 12, the defect was completely covered by compact lamellar bone, without signs of inflammation (Figure 14G–H).

## 4. Discussion

Magnesium-based biomaterials are considered promising alternatives to permanent metallic implants because they can provide temporary mechanical support and gradually degrade after implantation. Their degradation products can also influence the local biological response and bone formation [34,35]. In the present study, MRI 201s and ZMX 410 were evaluated from a material and biological perspective, including microstructural characterization, osteoblast viability, antimicrobial activity, subcutaneous tissue response, and bone regeneration.

The microstructural analysis showed clear differences between the two alloys. MRI 201s presented a more refined and homogeneous α-Mg microstructure, with globular and acicular secondary phases distributed within the grains and at the grain boundaries. XRD identified α-Mg, Mg–Zn–Nd phases, and the W-phase (Mg_3_Zn_3_Y_2_), while the I-phase was not detected. The formation and distribution of Mg–Zn–Y phases are influenced by the Zn/Y ratio and processing conditions, as also reported for other Mg–Zn–Y-containing alloys [36,37]. In ZMX 410, α-Mg and Mg_6_Zn_3_Ca_2_ were identified by XRD, while SEM–EDS also showed Ca- and Zn-rich secondary phases and Mn-rich regions. Similar phase distributions have been reported in recent Mg–Zn–Ca–Mn alloys, where changes in Zn, Ca, and Mn content influenced the microstructure, secondary-phase distribution, and corrosion behavior [29,38,39,40]. These differences between MRI 201s and ZMX 410 may therefore contribute to their different degradation and biological responses.

The cell culture results showed good cytocompatibility for both alloys. After 24 h of exposure, osteoblast viability remained between 97% and 112% for MRI 201s and between 101% and 115% for ZMX 410, with no significant differences compared with untreated cells. Similar results have been reported for other biodegradable magnesium alloys tested both in vitro and in vivo [9,41]. Magnesium ions released during degradation can support osteoblast activity and osteogenic differentiation, although their effects depend on concentration and exposure conditions [42,43]. Zinc may also contribute to the biological response of ZMX 410 because it is involved in several processes related to bone formation [43]. However, since the increase above 100% viability observed in the present study was not statistically significant, it should not be interpreted as a confirmed proliferative effect.

Both alloys also showed antimicrobial activity, although the MIC values differed according to the tested microorganism. The lowest MIC values were recorded against P. aeruginosa, at 2.88 mg/mL for MRI 201s and 2.96 mg/mL for ZMX 410. Magnesium-based alloys can inhibit bacterial growth through several changes associated with degradation, particularly the increase in local pH and the release of Mg^2+^ and other alloying ions [44]. The antimicrobial response of ZMX 410 may also be influenced by its Zn-containing composition, since Zn-containing biodegradable alloys have shown antibacterial activity in previous studies [41,44].

A useful comparison can be made with the Mg–Zn–Ca alloy investigated by Zhang et al. [45], as suggested by the reviewer. The authors evaluated an Mg–2Zn–0.5Ca alloy (ZC21) in comparison with Mg–4Zn–1Sr and pure Mg. ZC21 showed lower bacterial adhesion than the other Mg-based groups and also produced better bone-healing results in a femoral defect model [45]. Although the composition, geometry, and antimicrobial methods differ from those used in the present study, this comparison is relevant to ZMX 410 because both materials contain Mg, Zn, and Ca. In another experimental study, Chen et al. [41] compared Mg–0.8Ca with Mg–0.8Ca alloys containing Zn and Ag and found differences in degradation, antibacterial activity, and bone formation, according to alloy composition. Together with our findings, these studies show that relatively small changes in alloying elements can alter both degradation and biological performance. The Zhang study indeed found reduced MRSA adhesion for ZC21 compared with Mg–Zn–Sr and pure Mg, together with better healing in its femoral defect model. Chen et al. likewise reported composition-dependent differences in corrosion, antibacterial activity, and osteogenic response among Mg–Ca and Mg–Ca–Zn–Ag alloys.

The lack of inhibitory activity against *C. parapsilosis* for both materials is also relevant. *C. parapsilosis* is able to adhere to artificial surfaces and form biofilms, which can reduce susceptibility to antimicrobial agents [3,46]. Therefore, the absence of activity against this species should be considered when evaluating the antimicrobial potential of the alloys. Further studies using biofilm models would be more relevant than planktonic MIC testing alone for assessing their possible role in preventing implant-associated fungal infections.

The subcutaneous implantation model showed a different evolution of the local response between the two materials. Both groups showed an acute inflammatory reaction during the first week. In the MRI 201s group, the total lesion score decreased from 20.25 at Week 1 to 4 at Week 8, with minimal inflammation and no necrosis at the final time point. In contrast, ZMX 410 decreased from 16.75 at Week 1 to 9 at Week 2, but the score subsequently increased to 10.75 at Week 4 and 12.75 at Week 8. The later ZMX 410 samples also contained lymphocytes, plasma cells, macrophages, fatty infiltration, and lipid-laden macrophages.

The early tissue reaction may be related, at least in part, to magnesium degradation. Corrosion of magnesium in an aqueous environment is associated with hydrogen formation and local changes in pH, and gas accumulation has also been reported in in vivo studies of magnesium implants [47,48,49]. However, the different evolution of the two groups suggests that alloy composition and degradation behavior also influenced the local response. Recent in vivo studies of rare-earth-containing magnesium alloys have shown that a more controlled and uniform degradation pattern can be associated with a favorable tissue response and bone formation [49]. In the present study, the lower lesion score of MRI 201s at Week 8 suggests better late local tolerance than ZMX 410 under the experimental conditions used. A recent Mg–Ca–La study similarly found slow, relatively uniform degradation together with good in vivo biocompatibility and bone growth around the implant.

Micro-CT showed progressive changes in the amount and organization of mineralized tissue within the bone defect. At the early time points, both groups showed discontinuous mineralized regions and limited trabecular structures. Mineralized tissue became more continuous at Weeks 2 and 4, while by Week 8 both groups showed a more organized structure and greater continuity with the adjacent bone. Both materials showed an increase in bone volume and the percentage of bone within the VOI over time. MRI 201s presented higher surface density at the early time points, followed by a decrease as bone volume increased, whereas ZMX 410 showed lower early bone formation but higher bone volume at the final evaluation.

These changes are consistent with recent in vivo studies showing progressive bone formation around biodegradable magnesium implants [32,33,50,51]. Liu et al. [50], for example, reported new bone formation around porous WE43 magnesium scaffolds, while Wang et al. [33] showed that scaffold architecture and pore size influenced degradation and new bone formation. This is relevant to the present study because the distribution, surface area, and geometry of the implanted material can influence both degradation and the resulting bone response. Recent experimental work on additively manufactured magnesium scaffolds also showed that pore architecture affects both degradation and osteogenic response in vivo.

Histopathology provided additional information that could not be obtained from micro-CT alone. In MRI 201s, trabecular-like bone completely bridged the defect by Week 4, followed by further periosteal and endosteal proliferation and, at the later evaluation, by compact lamellar bone without inflammation. ZMX 410 showed slower defect filling, with incomplete trabecular coverage at Week 4 and complete closure by compact lamellar bone at Week 8. Therefore, both materials allowed bone formation, but the temporal pattern differed between them.

The earlier bridging observed with MRI 201s may be related to the microstructure and degradation pattern of the alloy. Rare-earth elements are commonly added to magnesium alloys to modify their mechanical and corrosion properties [16,17]. In particular, recent experimental studies of Mg alloys containing rare-earth elements have reported controlled degradation together with good bone response in vivo [47,49]. In contrast, the Zn- and Ca-containing composition of ZMX 410 may contribute differently to degradation and mineralization. Experimental Mg–Zn–Ca alloys have shown that Zn and Ca content can modify corrosion behavior and bone response [29,41,45]. The present results therefore suggest that the two alloys may reach a similar final outcome through different degradation and bone-remodeling patterns. This interpretation should, however, be confirmed by direct measurements of degradation rate and ion release.

The clinical importance of these findings is mainly related to the possible use of biodegradable magnesium materials in bone defects where temporary support is required. A material that gradually degrades while new bone forms could reduce the need for later implant-removal surgery. The antimicrobial activity observed for both alloys may also be useful, especially because implant-associated infection remains an important complication in orthopedic surgery [44,52,53,54,55]. However, the present MIC results were obtained in vitro and should not be interpreted as evidence that these alloys can prevent infection in vivo. An infected bone-defect model would be required before any such conclusion could be made.

The different responses of MRI 201s and ZMX 410 are also relevant when considering future implant design. MRI 201s showed earlier bone bridging and a lower late subcutaneous inflammatory score, whereas ZMX 410 showed substantial mineralized tissue at the later evaluation. Depending on the intended clinical application, controlling the degradation rate may therefore be as important as increasing the initial osteogenic response. The use of these alloys as solid implants or porous scaffolds may also be more suitable than loose powder for clinical applications, as implant geometry can limit material migration and allow better control of mechanical stability and degradation [33,50].

### 4.1. Limitations and Future Research

The present study has several limitations.

The in vitro results of the present study demonstrated that MRI 201s and ZMX 410 alloys were well tolerated by osteoblast cultures, with high cell viabilities indicating the absence of cytotoxic effects. Nevertheless, a careful distinction between basic cytocompatibility and beneficial proliferative signals driven by the released Mg/Zn ions should be taken into consideration to avoid misleading results. The MTT in vitro characterization can be reinforced by other fluorescent microscopy and flow cytometry determinations. Similar findings from previous studies showed that magnesium-based materials support osteoblast proliferation and metabolic activity [41,46,56,57,58]. The biological response to magnesium alloys is largely attributed to the release of Mg^2+^ ions, which can enhance cell adhesion, proliferation, and differentiation through activation of integrin-mediated signaling pathways and downstream cascades such as ERK [59,60,61].

To overcome the intrinsic limitations of metabolic assays like MTT, future research perspectives will look to incorporate additional verification techniques to assess cellular behavior: live/dead fluorescence microscopy will visually confirm cell morphology and spatial distribution in the presence of alloy powders. Furthermore, multiparametric flow cytometry utilizing Annexin V/Propidium Iodide (PI) apoptosis assays will be implemented in upcoming cohorts. These complementary methodologies will dissociate absolute cell viability from metabolic flux, offering a comprehensive understanding of the cytocompatibility, cell proliferation dynamics, and signaling cascades triggered by novel Mg alloy formulations.

The experimental period was limited to short- and mid-term evaluation intervals and therefore does not fully characterize long-term material degradation and late bone remodeling. In addition, no conventional metallic or pure magnesium control group was included, as the main objective was the direct comparison of MRI 201s and ZMX 410 under identical experimental conditions.

The use of powdered biomaterials may have influenced material distribution within the defect and the definition of the micro-CT volume of interest. Furthermore, the biological evaluation was mainly based on histopathology and micro-CT analysis.

Future studies should include longer follow-up periods and complementary investigations, such as assessment of degradation and ion release, mechanical testing, and evaluation of solid implants or standardized porous structures [62,63].

### 4.2. Clinical Relevance

From a clinical perspective, the investigated alloys may have potential as biodegradable materials for bone repair. Their ability to support bone formation while gradually degrading could reduce the need for secondary implant removal procedures. The antimicrobial activity observed in vitro may represent an additional advantage for orthopedic applications, although further in vivo studies are required before clinical use can be considered.

## 5. Conclusions

The present study showed that both MRI 201s and ZMX 410 biodegradable magnesium alloys were cytocompatible and supported bone regeneration, confirming their potential for bone defect repair. The two alloys produced different biological responses, with MRI 201s showing a more favorable early tissue response and earlier bone formation, while ZMX 410 showed a slower regenerative pattern with continued bone remodeling at later stages. These findings indicate that alloy composition plays an important role in determining the local tissue response and bone regeneration associated with biodegradable magnesium materials. Further studies are needed to evaluate long-term degradation, bone remodeling, and the biological effects of degradation products.

## Figures and Tables

**Table 1:** Elemental composition of the investigated magnesium-based alloys.

Alloy		Composition (Mass Percents %)					
	Zn	Zr	Y	Nd	Ca	Mn	Mg
ZMX 410/Mg-Zn	4.3	-	-	-	0.3	0.62	Bal.
MRI 201s/Mg-Nd	0.3	0.6	2.10	3.2	-	-	Bal.

**Table 2:** Elemental Composition of the Identified Phases.

**Point**	**MRI 201s**				
	**O**	**Zn**	**Y**	**Nd**	**Mg**
1	1.91 ± 0.12	0.19 ± 0.05	1.28 ± 0.21	1.46 ± 0.14	95.16 ± 0.30
2	1.57 ± 0.13	1.06 ± 0.08	4.53 ± 0.55	9.96 ± 0.18	82.88 ± 0.29
3	2.38 ± 0.20	1.37 ± 0.19	4.99 ± 0.63	13.75 ± 0.21	77.51 ± 0.25
4	2.05 ± 0.23	1.41 ± 0.06	6.08 ± 0.84	12.00 ± 0.18	78.46 ± 0.27
5	1.62 ± 0.14	0.57 ± 0.07	0.00	1.62 ± 0.14	96.19 ± 0.30
6	2.13 ± 0.21	0.31 ± 0.07	0.00	1.20 ± 0.14	96.36 ± 0.29
7	1.47 ± 0.13	0.56 ± 0.08	0.00	2.16 ± 0.15	95.81 ± 0.27
**Point**	**ZMX 410**				
	**O**	**Zn**	**Ca**	**Mn**	**Mg**
1	1.84 ± 0.16	2.37 ± 0.17	0.00	0.59 ± 0.08	95.20 ± 0.29
2	1.78 ± 0.15	2.58 ± 0.17	0.00	0.57 ± 0.08	95.07 ± 0.27
3	1.02 ± 0.20	36.42 ± 0.39	8.92 ± 0.09	0.00	53.64 ± 0.21
4	1.44 ± 0.15	24.88 ± 0.28	5.49 ± 0.06	0.00	68.19 ± 0.22
5	1.50 ± 0.18	42.59 ± 0.43	9.41 ± 0.07	0.00	46.50 ± 0.20
6	1.34 ± 0.19	42.82 ± 0.43	9.24 ± 0.10	0.00	46.60 ± 0.20
7	0.00	1.70 ± 0.19	0.81 ± 0.04	30.83 ± 0.17	66.66 ± 0.23
8	0.00	1.19 ± 0.14	0.35 ± 0.03	44.96 ± 0.20	53.50 ± 0.21

**Table 3:** Minimum inhibitory concentration (mg dry weight/mL).

	Gram (^+^) Bacteria			Gram (^−^) Bacteria			Fungi		
Samples	*S. aureus*	*S. epidermidis*	*C. parapsilosis*	*C. albicans*	*S. enterica*	*P. aeruginosa*	*E. coli*	*A. niger*	*A. bransiliensis*
MRI 201s (92.15 mg/mL)	46.07	46.07	n.b.	11.51	46.07	2.88	23.03	23.03	23.03
ZMX 410 (95 mg/mL)	n.b.	47.50	n.b.	11.87	47.50	2.96	23.75	11.87	23.75
Gentamicin (0.4 mg/mL)	0.0004	0.0016	-	-	0.0016	0.0001	0.0063	-	-
Fluconazole (1.5 mg/mL)	-	-	0.188	0.750	-	-	-	0.750	0.750

n.b.—no bioactivity. type of bacteria: Gram-positive (+) or Gram-negative (−).

**Table 4:** Comparative Lesion Scores in subcutaneous implantation of the experimental alloys (ISO 10993-6).

		Inflammation							Neovascularization	Fibrosis	Fatty Infiltration	Subtotal	Total (Sum)
		Acute		Chronic									
		Necrosis	PMN	Lymphocytes	Plasma Cells	Macrophage	Giant Cells	Subtotal					
**MRI 201s**	Week 1	3.25	4	1	0	2.25	2.75	**13.25**	4	2.25	0.75	**7**	**20.25**
	Week 2	0	0	1	0	3.25	2	**6.25**	1.5	1	0	**2.5**	**8.75**
	Week 4	0	1	1	0	3	1.25	**6.25**	3.25	1.25	0	**4.5**	**10.75**
	Week 8	0	1	0	0	1	0	**2**	1	1	0	**2**	**4**
**ZMX 410**	Week 1	3.75	4	0	0	2	1	**10.75**	2.75	3.25	0	**6**	**16.75**
	Week 2	0	0.5	1.5	0.5	2.5	1.5	**6.5**	1	1.5	0	**2.5**	**9**
	Week 4	0	0	3	1.5	3	1.25	**8.75**	1	1	0	**2**	**10.75**
	Week 8	1	0.75	1.75	2.5	2.5	0.75	**9.25**	1.25	1.75	0	**3**	**12.75**

## Data Availability

The original contributions presented in the study are included in the article, further inquiries can be directed to the corresponding author.

## References

[1] Aikin M., Shalomeev V., Kukhar V., Kostryzhev A., Kuziev I., Kulynych V., Dykh O., Dytyniuk V., Shapoval O., Zagorskis A. (2025). Recent advances in biodegradable magnesium alloys for medical implants: Evolution, innovations, and clinical translation. Crystals.

[2] Antoniac I., Adam R., Biță A., Miculescu M., Trante O., Petrescu I.M., Pogărășteanu M. (2021). Comparative assessment of in vitro and in vivo biodegradation of Mg-1Ca magnesium alloys for orthopedic applications. Materials.

[3] Niranjan C.A., Raghavendra T., Rao M.P., Siddaraju C., Gupta M., Jain V.K.S., Aishwarya R. (2023). Magnesium alloys as extremely promising alternatives for temporary orthopedic implants—A review. J. Magnes. Alloys.

[4] Wang J., Dou J., Wang Z., Hu C., Yu H., Chen C. (2022). Research progress of biodegradable magnesium-based biomedical materials: A review. J. Alloys Compd..

[5] Singh N., Batra U., Kumar K., Ahuja N., Mahapatro A. (2023). Progress in bioactive surface coatings on biodegradable Mg alloys: A critical review towards clinical translation. Bioact. Mater..

[6] Nasr Azadani M., Zahedi A., Bowoto O.K., Oladapo B.I. (2022). A review of current challenges and prospects of magnesium and its alloy for bone implant applications. Prog. Biomater..

[7] Lu Y., Deshmukh S., Jones I., Chiu Y.-L. (2021). Biodegradable magnesium alloys for orthopaedic applications. Biomater. Transl..

[8] Bonithon R., Lupton C., Roldo M., Dunlop J.N., Blunn G.W., Witte F., Tozzi G. (2023). Open-porous magnesium-based scaffolds withstand in vitro corrosion under cyclic loading: A mechanistic study. Bioact. Mater..

[9] Li D., Zhang D., Yuan Q., Liu L., Li H., Xiong L., Guo X., Yan Y., Yu K., Dai Y. (2022). In vitro and in vivo assessment of the effect of biodegradable magnesium alloys on osteogenesis. Acta Biomater..

[10] Zhang Z.Q., Yang Y.X., Li J.A., Zeng R.C., Guan S.-K. (2021). Advances in coatings on magnesium alloys for cardiovascular stents: A review. Bioact. Mater..

[11] Yao X., Tang J., Zhou Y., Atrens A., Dargusch M.S., Wiese B., Ebel T., Yan M. (2021). Surface modification of biomedical Mg-Ca and Mg-Zn-Ca alloys using selective laser melting: Corrosion behaviour, microhardness and biocompatibility. J. Magnes. Alloys.

[12] Ghorbani F., Mirzadeh H., Dehghanian C., Emamy M. (2023). Mechanical properties and corrosion behavior of biodegradable Mg–0.5Zr–0.5Ca–xZn magnesium alloys. Adv. Eng. Mater..

[13] Luo Y., Wang J., Ong M.T.Y., Yung P.S.-H., Wang J., Qin L. (2021). Update on the research and development of magnesium-based biodegradable implants and their clinical translation in orthopaedics. Biomater. Transl..

[14] Akbarzadeh F.Z., Sarraf M., Ghomi E.R., Kumar V.V., Salehi M., Ramakrishna S., Bae S. (2024). A state-of-the-art review on recent advances in the fabrication and characteristics of magnesium-based alloys in biomedical applications. J. Magnes. Alloys.

[15] Zhang T., Wang W., Liu J., Wang L., Tang Y., Wang K. (2022). A review on magnesium alloys for biomedical applications. Front. Bioeng. Biotechnol..

[16] Kumar A., Choudhari A., Gupta A.K., Kumar A. (2024). Rare-earth based magnesium alloys as a potential biomaterial for the future. J. Magnes. Alloys.

[17] Li H., Wang P., Lin G., Huang J. (2021). The role of rare earth elements in biodegradable metals: A review. Acta Biomater..

[18] Nie S., Chen J., Liu C., Zhou C., Zhao J., Wang Z., Sun J., Huang Y. (2023). Effects of extract solution from magnesium alloys supplemented with different compositions of rare earth elements on in vitro epithelial and osteoblast progenitor cells. Front. Bioeng. Biotechnol..

[19] Weng W., Biesiekierski A.A., Li Y., Dargusch M., Wen C. (2021). A review of the physiological impact of rare earth elements and their uses in biomedical Mg alloys. Acta Biomater..

[20] Roh H.-J., Park J., Lee S.-H., Kim D.-H., Lee G.-C., Jeon H., Chae M., Lee K.-S., Sun J.-Y., Lee D.-H. (2022). Optimization of the clinically approved Mg-Zn alloy system through the addition of Ca. Biomater. Res..

[21] Bexiga N.M., Alves M.M., Taryba M.G., Pinto S.N., Montemor M.F. (2022). Early biomimetic degradation of Mg-2Ca alloy reveals the impact of β-phases at the interface of this biomaterial on a micro-scale level. Corros. Sci..

[22] Asadollahi M., Gerashi E., Alizadeh R., Mahmudi R. (2022). Effect of Zn content and processing route on the microstructure, mechanical properties, and bio-degradation of Mg–Zn alloys. J. Mater. Res. Technol..

[23] Suh J.S., Ha H.-Y., Suh B.-C., Kang J.-W. (2023). Determination of optimum Zn content for Mg–xZn–0.5Mn–0.5Sr alloy in terms of mechanical properties and in vitro corrosion resistance. Met. Mater. Int..

[24] Hu Y., Dong D., Wang X., Chen H., Qiao Y. (2021). Synthesis and properties of Mg-Mn-Zn alloys for medical applications. Materials.

[25] Manescu (Paltanea) V., Antoniac A., Paltanea G., Antoniac I., Pall E., Moraru M.C., Dreanca A.I., Sevastre B., Stefanoiu R., Ciocoiu R. (2026). Biofunctional testing of a degradable implant made by Mg-Nd and Mg-Zn alloys used for bone defects. Biomimetics.

[26] Manescu (Paltanea) V., Antoniac A., Moraru M.C., Antoniac I., Cotrut C.M., Gradinaru S., Dreanca A.I., Sevastre B., Pop R., Tabaran F.A. (2025). Suitability of Mg-Nd and Mg-Zn alloys to obtain biodegradable structures for bone defects. J. Funct. Biomater..

[27] Lin T., Wang X., Jin L., Li W., Zhang Y., Wang A., Peng J., Shao H. (2021). Manufacturing of porous magnesium scaffolds for bone tissue engineering by 3D gel-printing. Mater. Des..

[28] Yin W., Briffod F., Shiraiwa T., Enoki M. (2022). Mechanical properties and failure mechanisms of Mg–Zn–Y alloys with different extrusion ratio and LPSO volume fraction. J. Magnes. Alloys.

[29] Istrate B., Munteanu C., Bălțatu M.-S., Cimpoeșu R., Ioanid N. (2023). Microstructural and electrochemical influence of Zn in MgCaZn biodegradable alloys. Materials.

[30] Huang S.-J., Wang C.-F., Subramani M., Fan F.-Y. (2023). Effect of ECAP on microstructure, mechanical properties, corrosion behavior, and biocompatibility of Mg-Ca alloy composite. J. Compos. Sci..

[31] Tomuleasa C.I., Foris V., Soritău O., Páll E., Fischer-Fodor E., Lung-Illes V., Brie I., Virág P., Perde-Schrepler M., Postescu I.D. (2009). Effects of 60Co Gamma-Rays on Human Osteoprogenitor Cells. Rom. J. Morphol. Embryol..

[32] Li W., Qiao W., Liu X., Bian D., Shen D., Zheng Y., Wu J., Kwan K.Y.H., Wong T.M., Cheung K.M.C. (2021). Biomimicking bone–implant interface facilitates the bioadaption of a new degradable magnesium alloy to the bone tissue microenvironment. Adv. Sci..

[33] Wang C., Liu J., Min S., Liu Y., Liu B., Hu Y., Wang Z., Mao F., Wang C., Ma X. (2023). The effect of pore size on the mechanical properties, biodegradation and osteogenic effects of additively manufactured magnesium scaffolds after high temperature oxidation: An in vitro and in vivo study. Bioact. Mater..

[34] Mao L., Hu Z., Song C. (2025). Biodegradable Mg-based medical devices: From passive support to active modulation. J. Magnes. Alloys.

[35] Chen Y., Zhang S., Bai J., Yang Y., Wang Y., Zhou Y., Jiang W., Wang J., Zhu J., Zhu C. (2024). Magnesium-based biomaterials for coordinated tissue repair: A comprehensive overview of design strategies, advantages, and challenges. J. Magnes. Alloys.

[36] Wang J., Wang J., Fu Q., Sheng K., Liu M., Sun Y., Mei D., Kou Y., Zhu S., Guan S. (2021). Microstructure, mechanical properties and corrosion fatigue behaviour of biodegradable Mg–Zn–Y–Nd alloy prepared by double extrusion. Corros. Eng. Sci. Technol..

[37] Hao J., Zhang J., Wang H., Cheng W., Bai Y. (2022). Microstructure and mechanical properties of Mg–Zn–Y–Mn magnesium alloys with different Zn/Y atomic ratio. J. Mater. Res. Technol..

[38] Cho D.H., Avey T., Nam K.H., Dean D., Luo A.A. (2022). In vitro and in vivo assessment of squeeze-cast Mg-Zn-Ca-Mn alloys for biomedical applications. Acta Biomater..

[39] Yu J., Wang L., Wang Y., Cao X., Li Y., Xing B., Lu L., Cheng W., Wang H., Shin K.S. (2022). Corrosion behavior of as-cast magnesium-4% zinc alloys in simulated body fluid solution: The influence of minor calcium and manganese addition. Mater. Werkst..

[40] Bazhenov V.E., Li A.V., Komissarov A.A., Koltygin A.V., Tavolzhanskii S.A., Bautin V.A., Voropaeva O.O., Mukhametshina A.M., Tokar A.A. (2021). Microstructure and mechanical and corrosion properties of hot-extruded Mg–Zn–Ca–(Mn) biodegradable alloys. J. Magnes. Alloys.

[41] Chen H., Yuan B., Zhao R., Yang X., Xiao Z., Antoniac A., Biță A.I., Zhu X., Antoniac V.I., Zhang X. (2022). Evaluation on the corrosion resistance, antibacterial property and osteogenic activity of biodegradable Mg-Ca and Mg-Ca-Zn-Ag alloys. J. Magnes. Alloys.

[42] Lin S., Yin S., Shi J., Yang G., Wen X., Zhang W., Zhou M., Jiang X. (2022). Orchestration of energy metabolism and osteogenesis by Mg2+ facilitates low-dose BMP-2-driven regeneration. Bioact. Mater..

[43] Chen Z., Zhang W., Wang M., Backman L.J., Chen J. (2022). Effects of zinc, magnesium, and iron ions on bone tissue engineering. ACS Biomater. Sci. Eng..

[44] Wang N., Ma Y., Shi H., Song Y., Guo S., Yang S. (2022). Mg-, Zn-, and Fe-based alloys with antibacterial properties as orthopedic implant materials. Front. Bioeng. Biotechnol..

[45] Zhang C., Lin J., Nguyen N.-Y.T., Guo Y., Xu C., Seo C., Villafana E., Jimenez H., Chai Y., Guan R. (2020). Antimicrobial bioresorbable Mg–Zn–Ca alloy for bone repair in a comparison study with Mg–Zn–Sr alloy and pure Mg. ACS Biomater. Sci. Eng..

[46] Zuo X.-S., Wang Q.-Y., Wang S.-S., Li G., Zhan L.-Y. (2024). The role of N-acetylcysteine on adhesion and biofilm formation of Candida parapsilosis isolated from catheter-related candidemia. J. Med. Microbiol..

[47] Zhang Y., Wang H., Kumazawa T., Ju D. (2023). In vivo degradation and bone reaction of long-term fixation with a magnesium alloy made by twin-roll casting in a rat femur model. Biomed. Mater. Eng..

[48] Ibrahim H., Billings C., Abdalla M., Korra A., Anderson D.E. (2023). In vivo assessment of high-strength and corrosion-controlled magnesium-based bone implants. Bioengineering.

[49] Ge W., Chen K., Tang H., Arken X., Zhang X., Gu X., Zhu C. (2022). Degradability and in vivo biocompatibility of micro-alloyed Mg-Ca-La alloys as orthopedic implants. Mater. Lett..

[50] Liu J., Liu B., Min S., Yin B., Peng B., Yu Z., Wang C., Ma X., Wen P., Tian Y. (2022). Biodegradable magnesium alloy WE43 porous scaffolds fabricated by laser powder bed fusion for orthopedic applications: Process optimization, in vitro and in vivo investigation. Bioact. Mater..

[51] Zhu Y., Jia G., Yang Y., Cheng J., Wang G., Jiang H., Wang H. (2023). Biomimetic porous magnesium alloy scaffolds promote the repair of osteoporotic bone defects in rats through activating the Wnt/beta-catenin signaling pathway. ACS Biomater. Sci. Eng..

[52] Allizond V., Comini S., Cuffini A.M., Banche G. (2022). Current knowledge on biomaterials for orthopedic applications modified to reduce bacterial adhesive ability. Antibiotics.

[53] Cui Z., Xiong Y., Liu Y., Zeng Y., Liu M., Liu X., Zeng Z., Zhang X., Xu S. (2025). Lowering creep rate in Mg-Zn-Ca magnesium alloy with hierarchical distribution of phases. Int. J. Plast..

[54] Nguyen N.Y.T., Grelling N., Wetteland C.L., Rosario R., Liu H. (2018). Antimicrobial activities and mechanisms of magnesium oxide nanoparticles against pathogenic bacteria, yeasts, and biofilms. Sci. Rep..

[55] Jin S.-E., Jin H.-E. (2021). Antimicrobial activity of zinc oxide nano/microparticles and their combinations against pathogenic microorganisms for biomedical applications: From physicochemical characteristics to pharmacological aspects. Nanomaterials.

[56] Luong A.D., Maruthapandi M., Gedanken A., Luong J.H.T. (2025). Rare-earth oxide nanoparticles: A new weapon against multidrug-resistant pathogens with potential wound healing treatment. Nanomaterials.

[57] Zhao Y., He P., Yao J., Li M., Wang B., Han L., Huang Z., Guo C., Bai J., Xue F. (2023). pH/NIR-responsive and self-healing coatings with bacteria killing, osteogenesis, and angiogenesis performances on magnesium alloy. Biomaterials.

[58] Sá Paiva S., Ferreira A., Pakenham E., Kaur K., Cavanagh B., O’Brien F.J., Murphy C.M. (2025). Magnesium ion-mediated regulation of osteogenesis and osteoclastogenesis in 2D culture and 3D collagen/nano-hydroxyapatite scaffolds for enhanced bone repair. J. Funct. Biomater..

[59] Cerqueira A., García-Arnáez I., Romero-Gavilán F., Azkargorta M., Elortza F., Martín de Llanos J.J., Carda C., Gurruchaga M., Goñi I., Suay J. (2022). Complex effects of Mg-biomaterials on the osteoblast cell machinery: A proteomic study. Biomater. Adv..

[60] Wang G., Luo J., Qiao Y., Zhang D., Liu Y., Zhang W., Liu X., Jiang X. (2022). AMPK/mTOR pathway is involved in autophagy induced by magnesium-incorporated TiO2 surface to promote BMSC osteogenic differentiation. J. Funct. Biomater..

[61] Branco J., Miranda I.M., Rodrigues A.G. (2023). Candida parapsilosis virulence and antifungal resistance mechanisms: A comprehensive review of key determinants. J. Fungi.

[62] Zhang Y., Xu J., Ruan Y.C., Yu M.K., O’Laughlin M., Wise H., Chen D., Tian L., Shi D., Wang J. (2016). Implant-derived magnesium induces local neuronal production of CGRP to improve bone-fracture healing in rats. Nat. Med..

[63] Hendea R.E., Raducanu D., Claver A., García J.A., Cojocaru V.D., Nocivin A., Stanciu D., Serban N., Ivanescu S., Trisca-Rusu C. (2023). Biodegradable magnesium alloys for personalised temporary implants. J. Funct. Biomater..

